# Carbon source-dependent metabolic states govern redox homeostasis and cofactor biosynthesis in *Propionibacterium freudenreichii*

**DOI:** 10.1128/msystems.01023-26

**Published:** 2026-09-28

**Authors:** Kirsi Savijoki, Bhawani Chamlagain, Minnamari Edelmann, Kaisa Hiippala, Paulina Deptula, Susanna Kariluoto, Tuula A. Nyman, Vieno Piironen, Pekka Varmanen

**Affiliations:** 1 Department of Food and Nutrition, University of Helsinki 202094, Helsinki, Finland; 2 Faculty of Pharmacy, Division of Pharmaceutical Chemistry and Technology, University of Helsinki 124454, Helsinki, Finland; 3 Faculty of Medicine, University of Helsinki 60655, Helsinki, Finland; 4 Department of Food Science, University of Copenhagen 378241, Frederiksberg, Denmark; 5 Department of Immunology, Faculty of Medicine, Institute of Clinical Medicine, University of Oslo 684092, Oslo, Norway; Medical Research Council Toxicology Unit, Cambridge, United Kingdom

**Keywords:** *Propionibacterium*, proteomics, carbon metabolism, amino acid biosynthesis, vitamin B12, cellular redox status, organic acids, oxidative stress

## Abstract

Microbial adaptation to fluctuating nutrient and oxygen conditions requires coordinated regulation of the metabolic network to maintain redox homeostasis within physicochemical and energetic constraints. While oxygen-dependent responses in *Propionibacterium freudenreichii* (PFR) have been characterized at the transcriptomic level, the role of carbon source in defining system-level metabolic states remains unclear. Here, we investigated carbon source-dependent metabolic reprogramming and cofactor biosynthesis in PFR strain DSM 20271^T^ using label-free quantitative proteomics integrated with physiological and metabolite analyses. Distinct carbon sources defined discrete metabolic states shaped by redox constraints, carbon routing, and biosynthetic prioritization. Lactate supported a comparatively balanced physiological state characterized by enhanced respiratory metabolism, amino acid biosynthesis, and riboflavin metabolism, enabling high specific vitamin B12 yields (~100 µg g^−1^ wet biomass). In contrast, hexose metabolism (glucose and fructose) imposed a redox-constrained state marked by upregulation of transport systems, glycolysis, and the pentose phosphate pathway, resulting in increased biomass but reduced biosynthetic efficiency. A defining feature of the hexose-driven state was activation of aspartate metabolism. Proteomic and metabolite data, together with functional assays, support a model in which aspartate is converted to fumarate and subsequently reduced to succinate consistent with an alternative electron sink that could facilitate NADH reoxidation under redox-constrained conditions. Together, these findings establish that carbon source shapes physiological state through carbon routing, redox homeostasis, and differential resource allocation, with cofactor biosynthesis emerging as a system-level property rather than a simple consequence of biosynthetic enzyme abundance.

**IMPORTANCE:**

*Propionibacterium freudenreichii* is used in food fermentations and is one of the few microorganisms able to synthesize biologically active vitamin B12, making it valuable for industry and biotechnology. Yet the metabolic principles governing its performance across growth conditions remain poorly understood. Here, we show that carbon source is a key determinant of metabolic state, dictating how cells resolve redox constraints and allocate biosynthetic resources. We identify an adaptation in sugar-grown cells in which aspartate metabolism provides a potential alternative electron sink to sustain redox balance under constrained conditions. By contrast, lactate supports a physiological state that promotes efficient vitamin B12 biosynthesis. These findings reveal a central role for carbon source in shaping metabolic configuration and identify redox balancing as a critical lever linking environmental inputs to biosynthetic output. This work provides mechanistic insight into redox-constrained metabolism and a framework for improving vitamin B12 production and other microbial bioprocesses.

## INTRODUCTION

*Propionibacterium freudenreichii* (PFR) is a metabolically versatile actinobacterium adapted to fluctuating anaerobic and microaerobic environments, where maintenance of redox homeostasis is central to growth and biosynthesis (1, 2). Its metabolism is organized around the Wood-Werkman cycle, which couples carbon flux from lactate and other carbon sources to propionate formation, ATP generation, and NADH reoxidation (3). More broadly, metabolic output in PFR is limited by the interplay between carbon flux distribution, redox homeostasis, and energy conservation, consistent with constraint-based models describing how physicochemical, thermodynamic, and stoichiometric limits shape metabolic fluxes (4–6). Carbon source availability strongly influences this organization, leading to substrate-dependent physiological states that reflect both genomic variability and regulatory flexibility (7, 8).

PFR is widely used in food fermentation and is one of the few microorganisms capable of synthesizing bioactive vitamin B12 (hereafter B12). Because plants generally do not contain B12, microbial production of the vitamin is important for human nutrition, particularly in plant-based diets (9, 10). Beyond its applied relevance, this species provides a useful model for investigating how physiological behavior emerges from interacting environmental and metabolic constraints. PFR can utilize a broad range of substrates, including carbohydrates and organic acids such as lactate (1, 7, 8). During anaerobic growth on lactate, carbon is channeled through the Wood-Werkman cycle, yielding propionate, acetate, and succinate. This pathway includes B12-dependent reactions, most notably methylmalonyl-CoA mutase, directly linking cofactor availability to flux distribution (1, 11). Changes in substrate utilization are therefore expected to influence both redox homeostasis and cofactor biosynthesis. Vitamin B12 biosynthesis in PFR proceeds through a complex *de novo* pathway encoded by approximately 30 genes (12). The pathway begins with uroporphyrinogen III and culminates in assembly of the corrin ring, which contains a central cobalt ion and distinct upper and lower ligands (13, 14). The lower ligand, 5,6-dimethylbenzimidazole (DMBI), is synthesized from riboflavin in an oxygen-dependent reaction, creating a requirement for both anaerobic and microaerophilic conditions during B12 production (15, 16). This hybrid pathway creates an inherent mismatch: conditions that favor B12-dependent metabolism do not necessarily coincide with those that maximize biosynthetic capacity (17).

Oxygen adds a further layer of complexity by requiring coordination between respiratory and fermentative metabolism to maintain redox homeostasis (18, 19). In PFR, oxygen influences energy metabolism, stress adaptation, respiration, and cofactor biosynthesis (19–21). Transcriptomic and surfaceome analyses have revealed extensive remodeling of transport systems and stress-response pathways during oxygen exposure (21). At the same time, emerging omics studies indicate that nutrient composition affects energy generation, precursor availability, and B12 production (22, 23). Notably, B12-dependent metabolism becomes increasingly important under low-oxygen conditions despite reduced biosynthetic capacity, revealing a mismatch between cofactor demand and synthesis (17). Together, these observations suggest that carbon source and oxygen jointly determine cellular physiology through a multilayered regulatory architecture integrating carbon metabolism, redox homeostasis, and cofactor biosynthesis (24).

Here, we investigated how carbon source influences cellular physiology, redox homeostasis, and cofactor biosynthesis in PFR strain DSM 20271^T^. Using label-free quantitative proteomics integrated with physiological and metabolite analyses, we compared cells grown on lactate, glucose, and fructose following an initial screening of five substrates. This approach revealed coordinated changes in transport systems, central carbon metabolism, amino acid pathways, and B12 biosynthesis. We further identified an aspartate-dependent fumarate-to-succinate pathway as a potential alternative electron sink supporting redox homeostasis during hexose metabolism. Together, these findings establish a systems-level framework linking substrate utilization to physiological adaptation and reveal broader principles of redox-constrained bacterial metabolism.

## MATERIALS AND METHODS

### PFR strain and culture conditions

The genome-sequenced PFR type strain DSM 20271^T^ (25) was obtained from DSMZ (Braunschweig, Germany). DSM 20271^T^ is lactose-negative, lacking the β-galactosidase activity required for lactose fermentation (7). An initial screen across five carbon sources (lactate, glucose, fructose, lactose, and inositol) confirmed this phenotype and identified lactate, glucose, and fructose as the substrates supporting the most distinct physiological responses (Table 1); these three were therefore selected for subsequent proteomic analysis. The strain was routinely maintained on YEL agar (1% [wt/vol] yeast extract, 0.5% [wt/vol] tryptone, and 0.8% [wt/vol] sodium DL-lactate) under anaerobic conditions at 30°C for 5–6 days and subcultured two to three times in liquid YEL medium (3–4 days per subculture). Prior to inoculation, cells were harvested, washed with 0.9% (wt/vol) NaCl, resuspended in an equal volume of 0.9% NaCl, and diluted 100-fold into YE medium (1% [wt/vol] yeast extract, 0.5% [wt/vol] tryptone, and 0.1 M K₂HPO₄/KH₂PO₄ buffer, pH 6.4) supplemented with 0.8% (wt/vol) sodium DL-lactate (LAC; Sigma-Aldrich), D-glucose (GLC; Merck), D-fructose (FRC; Merck), lactose (LAS; Sigma-Aldrich, Buchs, Switzerland), or inositol (INO; Sigma-Aldrich). Carbon sources were supplied at identical mass concentrations (0.8%, wt/vol) to facilitate comparison of substrate-specific physiological responses; carbon-equivalent normalization was not applied. To prevent Maillard reactions, media without carbohydrates were autoclaved separately (121°C, 15 min) and substrates were added aseptically. When indicated, cobalt chloride hexahydrate (5 mg/L; Sigma-Aldrich) was added to support B12 synthesis.

**TABLE 1:** Growth characteristics, production of metabolites (propionate and acetate), residual carbon in spent culture supernatants, and wet cell mass of *P. freudenreichii strain* DSM 20271^T^ grown in yeast extract-tryptone-based medium with different carbon sources

Parameter	Time (h)	Carbon source					
		Lactate	Glucose	Lactose^*a*^	Fructose	Inositol	No carbon
Optical density (OD_600_)	72	1.8 (0.01)	6.3 (0.18)	0.5 (0.02)	3.3 (0.06)	3.7 (0.01)	0.5
	168	2.4 (0.13)	6.1 (0.17)	1.5 (0.02)	4.7 (0.26)	3.7 (0.14)	1.5
pH	72^*b*^	>6.1	4.7	>6.1	5.6	4.7	>6.1
	168^*c*^	6.4 (0.05)	4.7 (0.01)	6.5 (0.03)	5.0 (0.01)	4.8 (0.01)	6.5
Propionate (mg/mL)	72	3.4 (0.06)	3.2 (0.08)	0.3 (0.01)	0.9 (0.07)	1.9 (0.05)	
	168	2.3 (0.11)	2.3 (0.06)	0.2 (0.00)	1.4 (0.17)	1.8 (0.13)	0.2
Acetate (mg/mL)	72	1.4 (0.04)	1.2 (0.02)	0.1 (0.01)	0.5 (0.03)	1.0 (0.03)	
	168	1.7 (0.11)	1.3 (0.03)	0.2 (0.02)	1.3 (0.10)	1.4 (0.12)	0.3
Residual carbon (mg/mL)	72	<0.10	0.10	8.3 (0.1)	5.0 (0.1)	1.3 (0.0)	
	168	<0.10	<0.10	7.4 (0.4)	1.1 (0.1)	0.0 (0.0)	
Wet cell mass (g/20 mL)	168	0.11 (0.00)	0.36 (0.04)	0.06 (0.01)	0.40 (0.03)	0.16 (0.00)	0.06

^*a*^ DSM 20271^T^ is lactose negative; growth on lactose-containing medium reflects utilization of yeast extract components rather than the added carbon source.

^*b*^ pH measured (indicative only) with pH paper.

^*c*^ pH measured with the pH meter.

Colony-forming ability of PFR cells in response to selected carbon sources (LAC, GLC, and FRC) was studied as follows. Cells were first cultured in YE medium supplemented with the indicated carbon source under the conditions described above. After four days (LAC- and GLC-grown cultures) and six days (FRC-grown cultures), cells were serially diluted (10^−4^ to 10^−8^) in phosphate-buffered saline (Dulbecco A, pH 7.3; Thermo Fisher Scientific). Five microliters of each dilution was spotted onto YE agar containing 0.8% (wt/vol) LAC, GLC, or FRC. Plates were incubated at 30°C under anaerobic (AnaeroGen 2.5 L sachets and anaerobic jar; <1% O_2_, ~10% CO_2_), semiaerobic (~20% O₂, 5% CO_2_), or aerobic (21% O_2_, <0.1% CO_2_) conditions for 4, 6, and 6–8 days, respectively. To assess the effect of aspartate on colony formation, cells were first cultivated in LAC-supplemented YE broth, serially diluted, and spotted onto YE agar containing LAC or FRC with or without 10 mM aspartate. Plates were incubated under the same atmospheric conditions as described above.

### Growth rate determination

Pre-cultures grown for 48 h were diluted 1:100 into fresh, unbuffered YE medium supplemented with the indicated carbon sources (0.8% [wt/vol]). Aliquots (300 µL) were dispensed into four interior wells of a Bioscreen C honeycomb plate per condition; edge wells were reserved for uninoculated medium controls. Plates were incubated at 30°C for 144 h in a Bioscreen C system (Oy Growth Curves Ab Ltd., Turku, Finland). Optical density (OD_600_) was measured every 4 h after 10 s of continuous shaking. At each time point, raw OD values were corrected by subtracting the mean absorbance of uninoculated control wells containing the corresponding medium. To restrict the analysis to the reported linear measurement range of the Bioscreen C, only raw OD₆₀₀ values ≤1.2 were included (26). Maximum specific growth rates (μ, h^−1^) were determined separately for the four parallel wells by linear regression of ln-transformed, control-corrected OD₆₀₀ values against time. Exponential-phase windows comprising three to four consecutive measurements were selected separately for each carbon source based on the growth curves and regression linearity (*R*^2^ ≥ 0.95 for each well). The resulting well-specific slopes are presented as mean ± SD.

### Cultivation for metabolite and vitamin analyses

Cells were cultivated in 20 mL YE medium supplemented with LAC, GLC, or FRC at 0.8% (wt/vol) (three biological replicates). Control cultures without cells and without added carbon sources were included. Cultures were incubated anaerobically at 30°C for 72 h, followed by 96 h under microaerophilic conditions (150 rpm shaking). Microaerophilic conditions were generated by briefly opening and closing tube caps under sterile conditions. Growth (OD_600_) and pH were measured at 72 h and 168 h. Cells were harvested at 168 h by centrifugation (12,000 × *g*, 10 min), washed with phosphate-buffered saline (PBS; pH 7.3), and stored at −20°C together with supernatants for subsequent analyses.

#### Vitamin B12 analysis

Vitamin B12 analysis with a UHPLC system was based on the method described previously (27). Cell pellets (0.05–0.3 g) were extracted in 10 mL acetate–NaOH buffer (pH 4.5) containing 1 mg sodium cyanide and incubated at 100°C for 30 min. After cooling, samples were centrifuged twice (6,900 × g, 10 min), adjusted to 25 mL, filtered (0.2 µm), and analyzed using a Waters UHPLC system with photodiode array detection (361 nm) and an HSS T3 C18 column. Gradient elution (0.32 mL/min) was performed using water/acetonitrile (5%–40%) containing 0.025% (vol/vol) trifluoroacetic acid. Vitamin B12 was quantified using a six-point external calibration curve of cyanocobalamin (Supelco, Bellefonte, PA, USA). Results were reported as volumetric B12 titer (µg mL^−1^) and biomass-specific B12 accumulation (µg g^−1^ fresh weight).

#### Riboflavin

Riboflavin (vitamin B2) levels in culture supernatants were determined by UHPLC with fluorescence detection as described previously (28). Samples were acid-extracted, enzymatically treated (Taka-diastase and β-amylase), filtered (0.2 µm), and analyzed using a BEH C18 column with excitation/emission at 432/520 nm. Riboflavin was quantified based on an external standard curve of riboflavin (0.01–1.0 ng µL^–1^).

#### Determination of sugars and organic acids

Sugars and organic acids were determined by an HPLC method as described previously (29). Samples were diluted (1:10), filtered (0.45 µm), and a chromatographic separation was performed using a Hi-Plex H column (Agilent, CA, USA; 300 × 6.5 mm) and 10 mM H_2_SO_4_ as a mobile phase (0.6 mL/min, 65°C). Acids were detected at 210 nm (UV), and sugars were detected by refractive index detection. External calibration curves (0.1–1 mg mL^−1^) were used for quantification.

#### Amino acid analysis

Free amino acids were determined on an Acquity UPLC system according to the manufacturer’s instructions for amino acid analysis (Waters Corporation, Milford, MA, USA). Analysis was carried out as reported by Kruk et al. (30) with small modifications. Cell-free supernatants were deproteinated using 3 kDa cutoff filters (10,000 × g, 15 min), diluted with borate buffer, and derivatized using the AccQ-Tag reagents (Waters). The chromatographic separation with a UHPLC system was performed as described previously (31). Amino acids were quantified based on the internal standard method using L-norvaline as an internal standard. A six-point calibration curve for each amino acid was established by calculating the ratio of peak area to the area of norvaline.

#### Statistical analysis

Statistical analyses were performed using SPSS v24.0 (IBM). Differences between conditions were evaluated using unpaired *t*-tests with Bonferroni correction. Significance thresholds are indicated in figure legends.

### Label-free quantitative (LFQ) proteomics

#### Extraction of proteins

PFR cells were grown in five biological replicates on LAC, GLC, or FRC under the conditions described above and harvested at mid-exponential growth, as established from preliminary growth curves obtained under the same cultivation conditions (Fig. S1A). Harvest times and corresponding OD₆₀₀ values were LAC, 20 h and 0.54 ± 0.03; GLC, 24 h and 0.50 ± 0.01; and FRC, 40 h and 0.66 ± 0.01. Cells were collected by centrifugation (5,000 × g, 15 min, 4°C), washed twice with ice-cold TEAB (pH 8.5) to remove loosely bound extracellular proteins (32), heated at 95°C, and stored at −80°C. For protein extraction, frozen cells were thawed at room temperature, suspended in 0.1% RapiGest SF (Waters Corp.) in 100 mM TEAB (pH 8.5), and transferred to FastPrep tubes containing ~50 µL glass beads (106 µm in diameter, Sigma Aldrich). Cells were disrupted in a FastPrep-24 (MP Biomedicals) at setting 6.5 for 4 × 30 s, centrifuged to pellet debris and beads, and frozen again at −80°C. After thawing, samples were heated to 70°C, cooled to room temperature, and subjected to a second round of FastPrep disruption. Then, 250 µL of 0.2% RapiGest in 100 mM TEAB (pH 8.0) was added, and samples were incubated at room temperature for 2–3 h with frequent mixing. Cell-free extracts were recovered by centrifugation (5000 × g, 5 min, RT), and protein concentration was measured using a NanoDrop 2000/2000c spectrophotometer (Thermo Fisher).

#### Proteomic analysis

Samples for LFQ proteomics were prepared using the PAC method (33) with modifications. Protein aggregation was induced from 10 µg of protein in RapiGest-buffer by adding acetonitrile to 70%, followed by capture on 10 µL MagReSyn Amine magnetic beads (LabLife Nordic AB, Finland). Bead-bound proteins were washed with 100% acetonitrile and 70% ethanol and briefly air-dried. For on-bead digestion, beads were submerged in 50 mM Tris-HCl (pH 8.0), proteins were reduced with 10 mM DTT for 30 min at 37°C, alkylated with 20 mM iodoacetamide for 30 min at room temperature, and quenched with 20 mM DTT. Proteins were digested overnight at 37°C with Trypsin Platinum (1:10, enzyme:protein) and rLysC (1:50) (Promega). After bead separation, peptides were acidified with 0.1% formic acid, purified using C18 microcolumns (Millipore), dried, resuspended in 0.1% formic acid, and analyzed by LC-MS/MS on a nanoElute UHPLC coupled to a timsTOF fleX mass spectrometer with a CaptiveSpray source (Bruker Daltonics, Bremen, Germany). Peptides were separated with a 60 min gradient at 300 nL/min, and data were acquired in DDA-PASEF mode using 10 MS/MS scans per cycle.

Raw MS files were processed in MaxQuant v2.0.1.0 (34, 35) against the NZ_CP010341.1 database (25) containing 2,320 protein sequences. Carbamidomethylation of cysteine was set as a fixed modification, while methionine oxidation and protein N-terminal acetylation were included as variable modifications. Peptide mass tolerance was set to 20 ppm for the first search and 10 ppm for the main search. Trypsin without proline restriction was specified with up to two missed cleavages. Protein identification required at least one unique or razor peptide, and peptide and protein FDRs were controlled at 1% using reversed sequences. The raw proteomics data have been deposited to the ProteomeXchange Consortium via the PRIDE (36) partner repository with the data set identifier PXD079229.

#### Proteome statistics

Downstream proteomic analyses were performed in Perseus v1.6.15.0 (37) using LFQ intensity values from the identified protein groups, with protein annotations confirmed by retrieving the protein sequences for the identified proteins from UniProtKB (38). Decoys, contaminants, and proteins identified only by site were excluded. Proteins detected in at least three of five replicates in at least one condition were retained; data were log2 transformed, and missing values were imputed from a normal distribution (width 0.3, downshift 1.8) and subjected to principal component analysis (PCA) to assess global proteomic variation and sample clustering between conditions. Z-score-normalized LFQ values were used for Euclidean hierarchical clustering and heatmap generation. Significant abundance changes were identified using multi-sample ANOVA with permutation-based FDR correction at 0.05, followed by Tukey’s HSD *post hoc* testing.

## RESULTS

### Growth dynamics and metabolite formation were strongly carbon source-dependent

**Fig 1:**
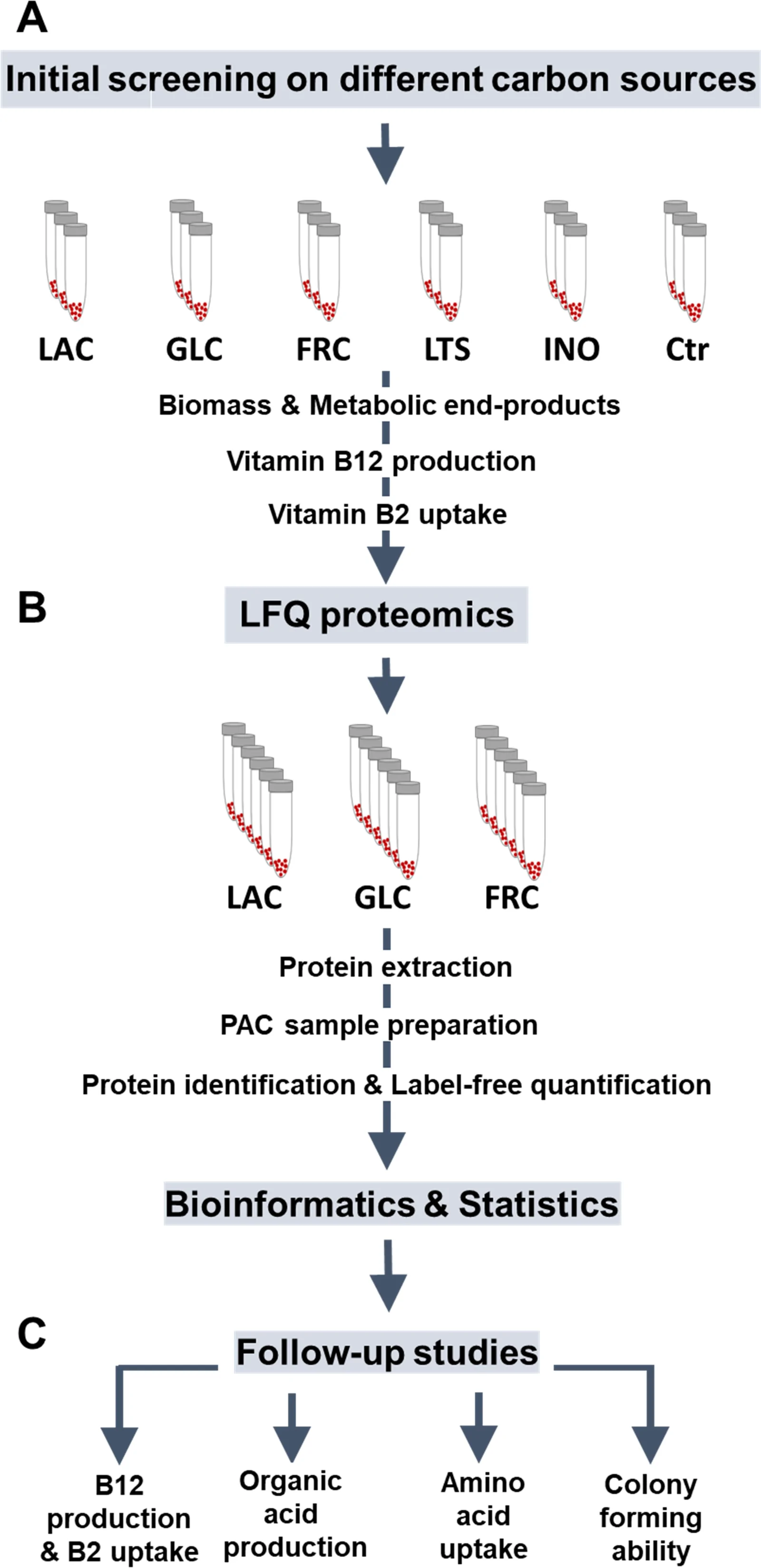
Experimental workflow for evaluating carbon source-dependent effects on growth, metabolism, cofactor production, and proteome-wide adaptation in *P. freudenreichii* DSM 20271^T^. (**A**) Initial screening across five carbon sources—lactate (LAC), glucose (GLC), fructose (FRC), lactose (LAS), and inositol (INO)—was used to assess biomass formation, metabolic end-products, vitamin B12 accumulation, and riboflavin uptake. (**B**) Based on the screening results, LAC-, GLC-, and FRC-grown cultures were selected for label-free quantitative (LFQ) proteomics. Cells were processed by protein extraction, PAC-based sample preparation, protein identification, and label-free quantification, followed by bioinformatics and statistical analysis. (**C**) Proteomic and phenotypic findings informed an integrated physiological characterization of vitamin B12 accumulation and riboflavin uptake, organic acid production, amino acid consumption, and colony-forming ability. Ctr indicates control medium without added carbon substrate.

**Fig 2:**
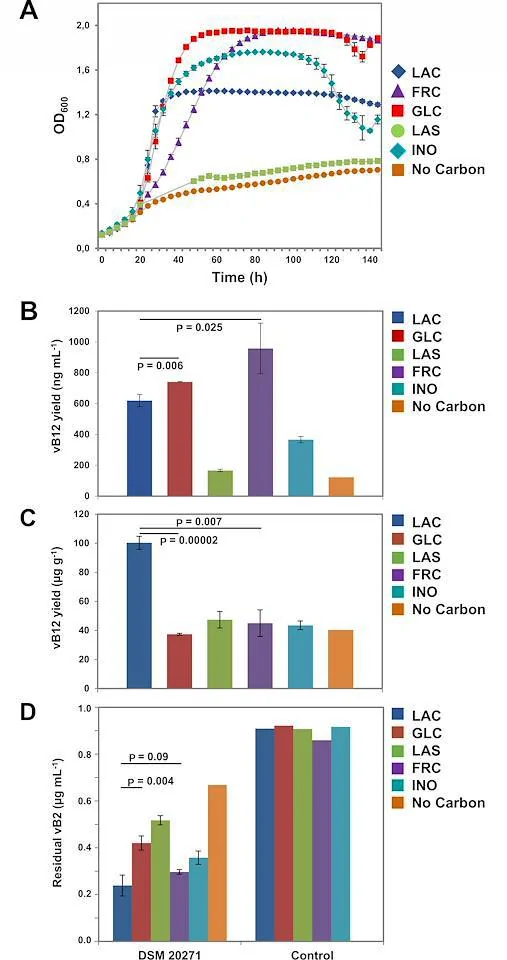
Growth characteristics of *P. freudenreichii* DSM 20271^T^ and carbon source-dependent vitamin B12 accumulation and riboflavin uptake. (**A**) Growth of *P. freudenreichii* DSM 20271^T^ in yeast extract–tryptone (YE)-based medium supplemented with different carbon sources, monitored by optical density at 600 nm in a Bioscreen C system at the indicated time points. (**B**) Volumetric vitamin B12 titer (ng mL^−1^ of total culture volume) in YE medium containing different carbon sources after fermentation by *P. freudenreichii* DSM 20271^T^. (**C**) Biomass-specific vitamin B12 content (µg g^−1^ wet cell biomass) of *P. freudenreichii* DSM 20271^T^ grown in YE with different carbon sources. Error bars in panels **B and C** indicate the standard deviation of three independent replicate cultures. Statistical significance was assessed by unpaired *t*-test with equal variance assumption; *P*-values for indicated comparisons are shown. (**D**) Residual vitamin B2 (riboflavin) in spent culture supernatants of *P. freudenreichii* DSM 20271^T^ grown in YE medium supplemented with different carbon sources after 168 h at 30°C. Initial riboflavin concentration in fresh YE medium was 0.9–1.0 µg mL^−1^, as indicated by uninoculated control cultures incubated under identical conditions. Statistical significance between lactate and the indicated conditions is shown.

Figure 1 summarizes the experimental workflow designed to assess how carbon source shapes growth, metabolism, cofactor production, and proteome-wide adaptation in PFR DSM 20271. An initial screen across five carbon sources evaluated biomass formation, metabolic end-products, B12 production, and riboflavin uptake. Based on this screen, three representative conditions—lactate (LAC), glucose (GLC), and fructose (FRC)—were selected for LFQ proteomics, followed by bioinformatics and statistical analysis. Building on these proteomic findings, we then characterized B12 and riboflavin accumulation, organic acid production, amino acid consumption, and colony-forming ability across conditions to provide an integrated physiological account of carbon source-dependent metabolic adaptation. Growth kinetics differed markedly across substrates (Fig. 2A). Based on linear regression of ln-transformed OD₆₀₀ values during exponential growth, LAC supported the highest specific growth rate (0.150 ± 0.008 h^−1^), whereas FRC showed the lowest (0.061 ± 0.004 h^−1^); the corresponding rate for GLC was 0.128 ± 0.010 h^−1^. LAC cultures reached stationary phase after ~50 h, whereas FRC cultures reached stationary phase after ~90 h. Despite slower growth, GLC and FRC cultures reached the greatest final cell densities (OD_600_ 6.1 ± 0.17 and 4.7 ± 0.26, respectively), compared with LAC (OD_600_ 2.4 ± 0.13) (Table 1). Because carbon sources were supplied at equal mass concentrations (0.8% [wt/vol]) rather than equimolar or carbon-equivalent amounts, the observed differences in biomass and metabolite yields reflect both substrate identity and differences in the total available carbon and reducing equivalents per unit volume; these contributions cannot be fully separated in the present experimental design. Biomass recovery after 168 h followed the same trend. Substrate consumption and pH profiles reflected these growth patterns. LAC and GLC were largely depleted during the anaerobic phase, whereas FRC was consumed more slowly. Higher biomass formation in hexose cultures coincided with medium acidification (pH 4.7–5.0), whereas LAC cultures maintained near-neutral pH (~6.4). During anaerobic growth, LAC and GLC cultures produced the highest levels of propionate and acetate, whereas FRC cultures produced substantially lower concentrations. Transition to microaerophilic conditions shifted fermentation toward higher acetate-to-propionate ratios in all cultures.

### Carbon source determines B12 titer and biomass-specific accumulation

Volumetric B12 titer varied strongly by carbon source (Fig. 2B). Without carbon supplementation, DSM 20271 produced 120 ng mL^−1^ B12. LAC and GLC increased yields to 619 and 741 ng mL^−1^, respectively, whereas FRC supported the highest volumetric titer (957 ng mL^−1^). In contrast, biomass-normalized values showed the opposite trend (Fig. 2C). LAC-grown cells exhibited the highest specific B12 content (~100 µg g^−1^ fresh biomass), whereas values for the other carbon sources remained below 50 µg g^−1^.

Because flavin cofactors derived from riboflavin participate in redox reactions associated with corrinoid biosynthesis and central carbon metabolism, residual riboflavin was monitored as an indicator of cofactor demand during B12 production. Residual riboflavin decreased during fermentation (Fig. 2D). LAC cultures showed the lowest residual levels (0.24 µg mL^−1^), indicating high net uptake from the medium, whereas FRC cultures retained higher levels (0.56 µg mL^−1^) despite higher volumetric B12 accumulation. Together, these findings show that carbon source influences both total B12 production and biomass-specific corrinoid accumulation, while also altering riboflavin utilization.

### Proteomic adaptation to lactate and hexose growth conditions

**Fig 3:**
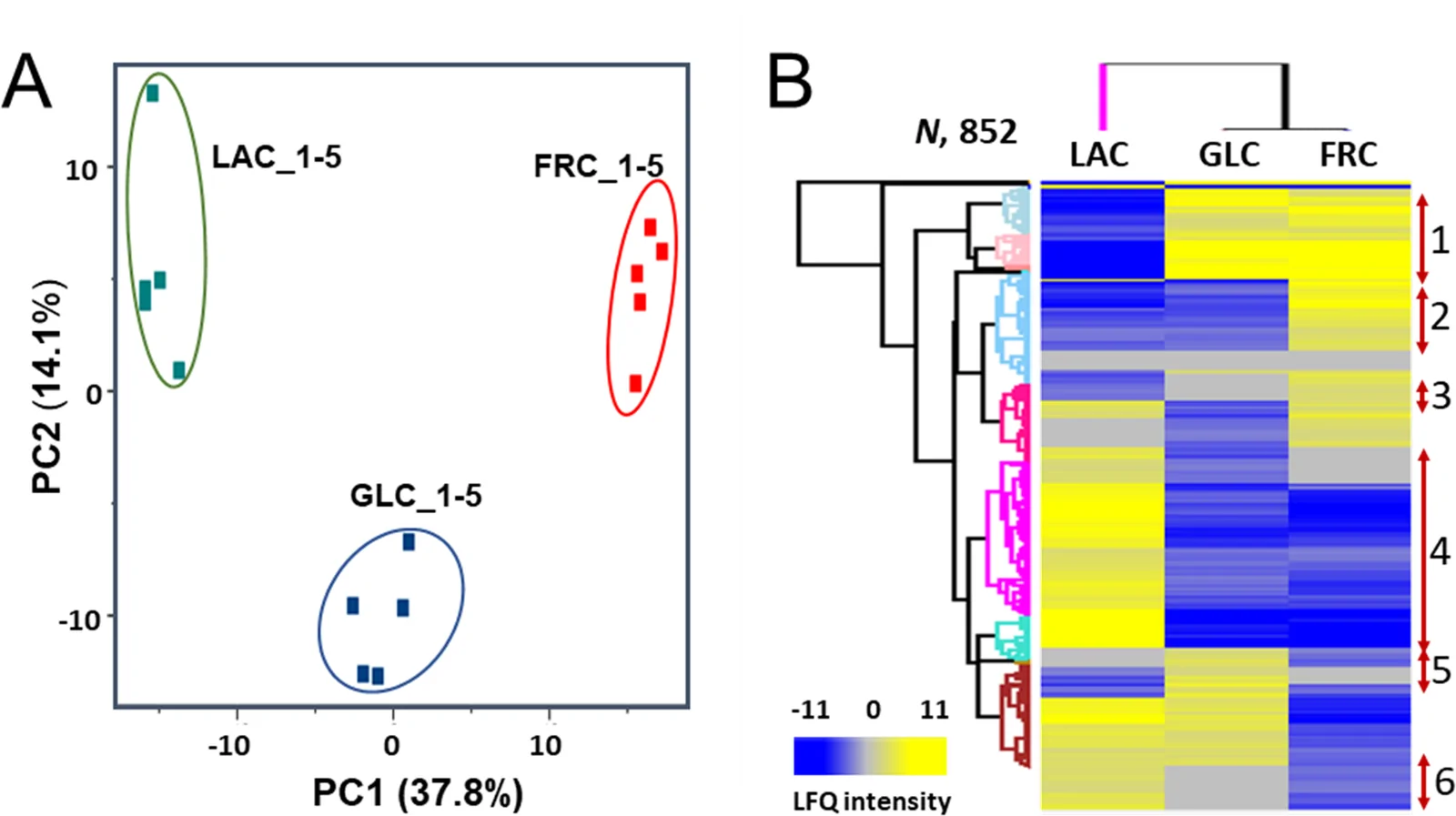
Multivariate analyses of LFQ proteomics data. (**A**) Principal component analysis (PCA) of all detected proteins based on LFQ intensities. The five biological replicates per condition cluster closely, indicating high reproducibility and clear separation of proteomes according to carbon source (LAC, GLC, FRC). (**B**) Hierarchical clustering of proteins with significant abundance differences across conditions. Proteome-wide ANOVA (permutation-based FDR = 0.05; *q* < 0.05), followed by post hoc Tukey’s HSD test, identified 851 differentially abundant proteins. Z-score-normalized LFQ intensities were subjected to Euclidean distance-based clustering, resulting in six distinct clusters that capture carbon source-dependent patterns and relationships within the LAC-, GLC-, and FRC-associated proteomes.

Label-free quantitative proteomic analysis identified 1,560 proteins across PFR cells grown under LAC, GLC, and FRC conditions (Table S1), with high reproducibility across biological replicates (Fig. S2). Multivariate analyses, including principal component analysis (Fig. 3A) and hierarchical clustering (Fig. 3B), showed clear group separation according to carbon source. Proteome-wide ANOVA following Perseus processing identified 851 proteins with significantly different abundances among growth conditions (*q* < 0.05) (Table S2). The greatest abundance differences are summarized in Table S3.

#### LAC-associated changes

In LAC-grown cells, proteins with increased abundance relative to GLC- and FRC-grown cells were mainly associated with respiratory metabolism, redox homeostasis, amino acid biosynthesis, transport, and stress adaptation (Table S3; *z*-score normalized abundance values; positive values indicate relative enrichment in the indicated condition). Enrichment of respiratory electron-transfer components, including succinate dehydrogenase/fumarate reductase subunits SdhB/FrdB and SdhA/FrdA, and anaerobic glycerol-3-phosphate dehydrogenase subunit GlpB, together with pyruvate oxidation enzymes dihydrolipoamide acetyltransferase AceF/DlaT and pyruvate dehydrogenase E1α subunit AceE/PdhE1 was consistent with increased investment in respiratory metabolism during growth on lactate. Increased abundance of thioredoxin, ferredoxin-nitrite reductase NirA, and Fe–S cluster assembly proteins SufD, SufS, and SufC further supported elevated redox and cofactor-related activity. LAC-grown cells also showed increased levels of the L-lactate permease LldP, ferrous iron transporter FeoA, and osmoprotectant transport systems, suggesting coordinated adaptation to lactate utilization and cofactor demand. In addition, higher abundance of molecular chaperones and replication-associated proteins, including GroEL, DnaK, RecA, and DnaA, pointed to increased biosynthetic activity and stress-responsive capacity in LAC-grown cells.

#### FRC-associated changes

FRC-grown cells showed a distinct enrichment of proteins relative to LAC-grown cells involved in fructose and glucose interconversion, sugar phosphate turnover, pentose metabolism, and carbohydrate storage. Increased abundance of phosphoglucomutase, glucose-6-phosphate isomerase, glucose-1-phosphate adenylyltransferase, fructose-bisphosphate aldolase (Fba), ribulokinase, and gluconokinase indicated enhanced metabolic flexibility during fructose utilization. In addition, enrichment of starch synthase and branching enzyme GlgB suggested a fructose-specific shift toward carbohydrate reserve metabolism. Proteins involved in phosphate and energy metabolism, including polyphosphate kinase 2 (PPK2), polyphosphate-glucose phosphotransferase, and phosphoenolpyruvate-protein phosphotransferase, were also increased, consistent with elevated energetic demand during rapid sugar phosphate turnover and carbohydrate catabolism.

#### Hexose-associated changes

**Fig 4:**
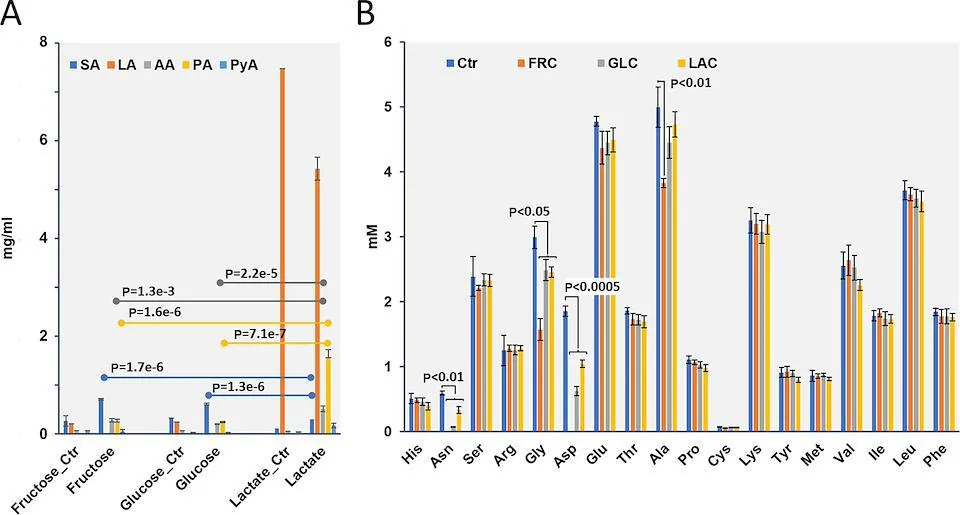
Organic acid production and amino acid consumption depend on carbon source. (**A**) Organic acid concentrations in cell-free culture supernatants of *P. freudenreichii* DSM 20271^T^ grown on fructose (FRC), glucose (GLC), or lactate (LAC), compared with uninoculated control medium (Ctr). Succinic acid (SA), lactic acid (LA), acetic acid (AA), propionic acid (PA), and pyruvic acid (PyA) were determined by HPLC after 168 h of cultivation. (**B**) Amino acid concentrations in spent culture supernatants following growth on FRC, GLC, or LAC, compared with uninoculated control medium. Amino acids were quantified after derivatization using UHPLC. Asparagine (Asn), aspartate (Asp), and glycine (Gly) showed marked depletion in hexose-grown cultures relative to LAC-grown cultures and uninoculated controls, consistent with increased amino acid utilization under hexose conditions. Statistical significance was determined using unpaired *t*-tests with Bonferroni correction; *P*-values for indicated comparisons are shown.

GLC- and FRC-grown cells showed strong enrichment of proteins relative to LAC-grown cells associated with carbohydrate uptake, central carbon metabolism, energy generation, and reductive metabolism. High abundance of the ABC transporter component YtfQ, inositol-3-phosphate synthase Ino1, pyruvate phosphate dikinase PpdK, and phosphogluconate dehydrogenase Gnd indicated increased carbohydrate uptake, glycolytic activity, and pentose phosphate pathway activity under both hexose conditions relative to LAC-grown cells. Additional enrichment of galactokinase GalK, aldose 1-epimerase GalM, NAD(P)^+^-dependent glycerol-3-phosphate dehydrogenase GlpD, transaldolase, and phosphoenolpyruvate-protein phosphotransferase further supported enhanced carbohydrate interconversion and redox-associated metabolism during growth on glucose and fructose relative to LAC-grown cells. Hexose-grown cells also showed increased abundance of proteins linked to overflow metabolism, alternative carbon utilization, and reductive metabolism, particularly under FRC conditions. Enrichment of aspartate ammonia-lyase AspA, alanine dehydrogenase, glycerate kinase, phosphoketolase-related proteins, and glyoxylate reductase suggested extensive redistribution of carbon and nitrogen metabolism under hexose conditions. Consistently, GLC- and FRC-grown cultures produced approximately two-fold higher succinate levels than LAC-grown cultures (*P* < 2.0 × 10^−6^) (Fig. 4A), supporting increased reductive TCA-associated flux during hexose metabolism.

Together, these findings indicate that lactate promotes a respiratory and stress-adapted proteomic state, whereas glucose and fructose favor rapid carbohydrate turnover, overflow metabolism, and extensive metabolic reorganization.

### Carbon source redirects amino acid and nitrogen metabolism

Proteomic analysis also revealed marked carbon source-dependent differences in amino acid and nitrogen metabolism (Table S3; *z*-score-normalized abundance values; positive values indicate relative enrichment in the indicated condition). LAC-grown cells showed increased abundance of proteins associated with amino acid biosynthesis, sulfur assimilation, and Fe–S cluster assembly relative to GLC- and FRC-grown cells. Among the most enriched proteins were arginine biosynthesis bifunctional protein ArgJ, ferredoxin-nitrite reductase NirA, ketol-acid reductoisomerase IlvC, argininosuccinate synthase, and acetylglutamate kinase, indicating enhanced branched-chain and arginine biosynthetic activity during growth on lactate. Increased abundance of sulfur-associated proteins, including sulfate adenylyltransferase subunits 1 and 2, phosphoadenosine phosphosulfate reductase, and cysteine synthase, together with Fe–S assembly proteins SufD, SufS, and SufC, further supported enhanced sulfur utilization and cofactor biosynthesis under lactate conditions.

In contrast, GLC- and FRC-grown cells displayed increased abundance of proteins associated with amino acid catabolism, carbon redistribution, and reductive metabolism relative to LAC-grown cells. Aspartate ammonia-lyase AspA was strongly enriched in GLC- and FRC-grown cells relative to LAC-grown cells. Additional enrichment of alanine dehydrogenase, aspartate racemase, glycerate kinase, and glyoxylate reductase suggested coordinated redistribution of carbon and nitrogen metabolism during growth on hexoses. Hexose-grown cells also showed elevated levels of phosphogluconate dehydrogenase Gnd, pyruvate phosphate dikinase PpdK, and NAD(P)^+^-dependent glycerol-3-phosphate dehydrogenase GlpD, consistent with elevated pentose phosphate pathway activity, increased NADPH demand, and enhanced reductive metabolism during growth on glucose and fructose.

### Hexose-associated amino acid depletion is linked to reduced aerobic viability

**Fig 5:**
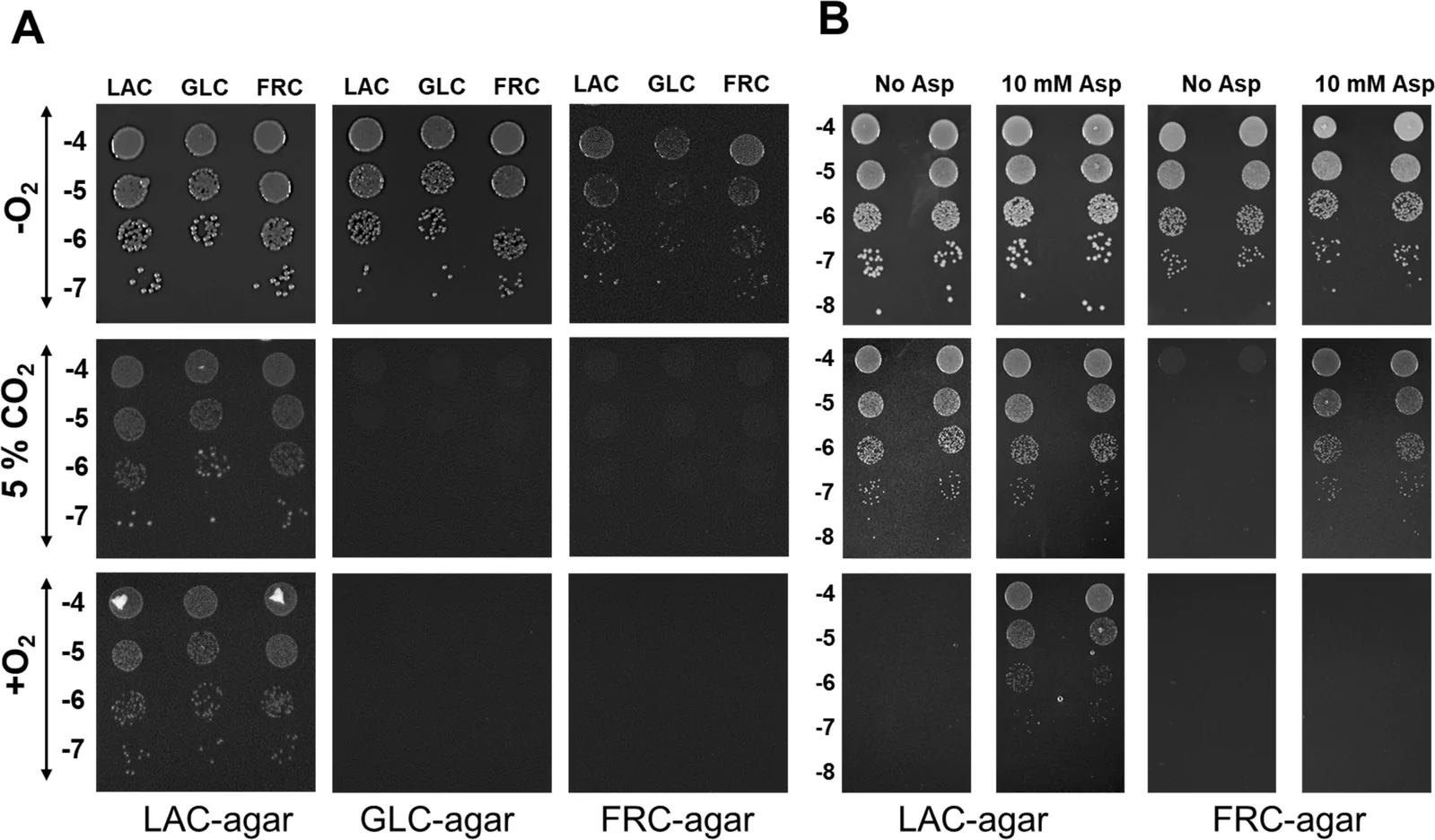
Carbon source- and oxygen-dependent colony-forming ability of *P. freudenreichii* DSM 20271^T^. (**A**) Colony formation of cells grown on lactate (LAC), glucose (GLC), or fructose (FRC) and spotted as serial dilutions (10^−4^ to 10^−7^/10^−8^) on YE agar containing the indicated carbon source. Plates were incubated at 30°C under anaerobic (−O_2_; 4 days), semiaerobic (5% CO_2_; 6 days), or aerobic (+O_2_; 8 days) conditions. Colony formation was comparable across preculture conditions when plated on LAC agar, whereas growth on GLC and FRC agar resulted in reduced colony formation, particularly under oxygen-exposed conditions. (**B**) Effect of aspartate supplementation on colony formation. Cells grown on LAC or FRC were spotted on YE agar containing the indicated carbon source with or without aspartate (10 mM). Anaerobic and semiaerobic plates were incubated as in panel **A**, whereas aerobic plates were incubated for 6 days. Aspartate supplementation restored colony formation on FRC agar, particularly under semiaerobic conditions, whereas only minor effects were observed on LAC agar. These results support a role for aspartate in maintaining growth and viability under redox-constrained conditions associated with fructose metabolism.

To validate the proteomic findings, amino acid profiles were analyzed during growth on different carbon sources. Asparagine, aspartate, and glycine were rapidly depleted in hexose-grown cultures (Fig. 4B), supporting increased amino acid utilization under these conditions. Although colony-forming ability in liquid cultures remained comparable among treatments, colony formation on solid media was strongly carbon source-dependent. GLC- and FRC-containing plates showed markedly reduced colony formation, particularly under semiaerobic and aerobic conditions (Fig. 5A). Notably, aspartate supplementation restored colony formation on FRC plates (Fig. 5B), linking hexose-associated amino acid depletion to impaired aerobic viability. Thus, hexose metabolism was associated with increased amino acid consumption, particularly of aspartate and asparagine, together with reduced colony-forming ability under oxygen-exposed conditions.

### Aspartate metabolism drives succinate accumulation as an alternative electron sink

**Fig 6:**
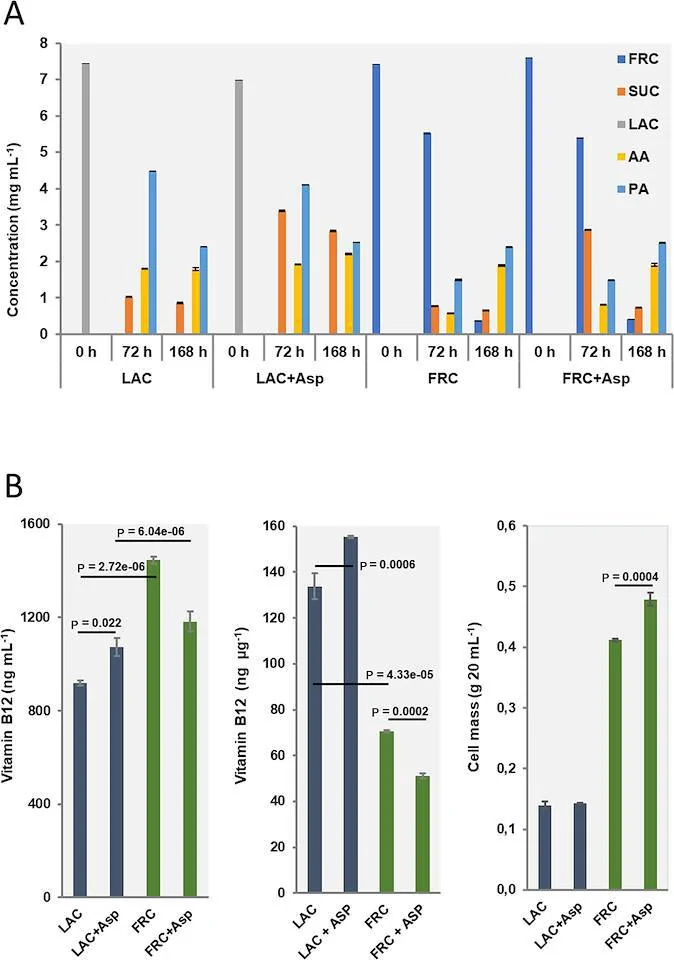
Aspartate supplementation reshapes redox metabolism and vitamin B12 accumulation. (**A**) Time-resolved metabolite profiles in *P. freudenreichii* DSM 20271^T^ cultures grown on lactate (LAC) or fructose (FRC), with or without aspartate supplementation (10 mM). Residual concentrations of fructose and lactate (carbon substrates) and fermentation products succinate (SUC), acetate (AA), and propionate (PA) were measured at 0, 72, and 168 h. Aspartate supplementation led to increased succinate accumulation during anaerobic growth, particularly in FRC cultures, consistent with activation of an aspartate-linked fumarate–succinate pathway. Residual substrate consumption and organic acid profiles differed between LAC and FRC conditions. (**B**) Effects of aspartate supplementation on volumetric vitamin B12 titer (left), biomass-specific vitamin B12 content (middle), and biomass (right) in LAC- and FRC-grown cultures. Aspartate supplementation increased volumetric vitamin B12 titer and biomass-specific content in LAC cultures but reduced both in FRC cultures, while increasing cell biomass under FRC conditions. Statistical significance was determined using unpaired *t*-tests with Bonferroni correction; *P*-values for indicated comparisons are shown.

Because proteomic analysis revealed strong enrichment of AspA together with increased succinate production under hexose conditions, we investigated whether aspartate contributes to reductive metabolism through fumarate-dependent succinate formation. To test this, cultures were supplemented with 10 mM aspartate, which triggered a 3- to 4-fold increase in succinate accumulation during anaerobic growth in both FRC + ASP and LAC + ASP cultures (0.29 and 0.34 mg mL^−1^, respectively) compared with unsupplemented cultures (~0.08–0.10 mg mL^−1^) (Fig. 6A). This is consistent with rapid aspartate depletion and strong upregulation of aspartate ammonia-lyase, indicating active conversion of aspartate to fumarate and subsequent reduction to succinate. Following the shift to microaerophilic conditions, succinate was rapidly consumed in FRC + ASP cultures but remained stable in LAC + ASP cultures. Aspartate supplementation did not significantly affect propionate or acetate production. Together, these findings support the interpretation that aspartate primarily contributes to redox homeostasis rather than serving as a major carbon source, and that this role is particularly important for aerobic viability during hexose metabolism.

### Aspartate supplementation exerts opposing effects on B12 production

Because B12 biosynthesis depends on both precursor availability and cellular redox balance, we next examined whether aspartate supplementation differentially affects B12 production during lactate and fructose metabolism. Aspartate supplementation increased B12 production in LAC-grown cells, improving both volumetric and specific yields by 16%–17%, but reduced production in FRC-grown cultures by 18% volumetrically and 27% on a biomass-specific basis (Fig. 6B). These opposing responses suggest that the metabolic fate of supplemented aspartate differs between carbon sources, potentially supporting B12 precursor supply during lactate metabolism while contributing preferentially to redox balancing during fructose metabolism.

### Carbon source differentially affects corrinoid- and riboflavin-associated pathways

**Fig 7:**
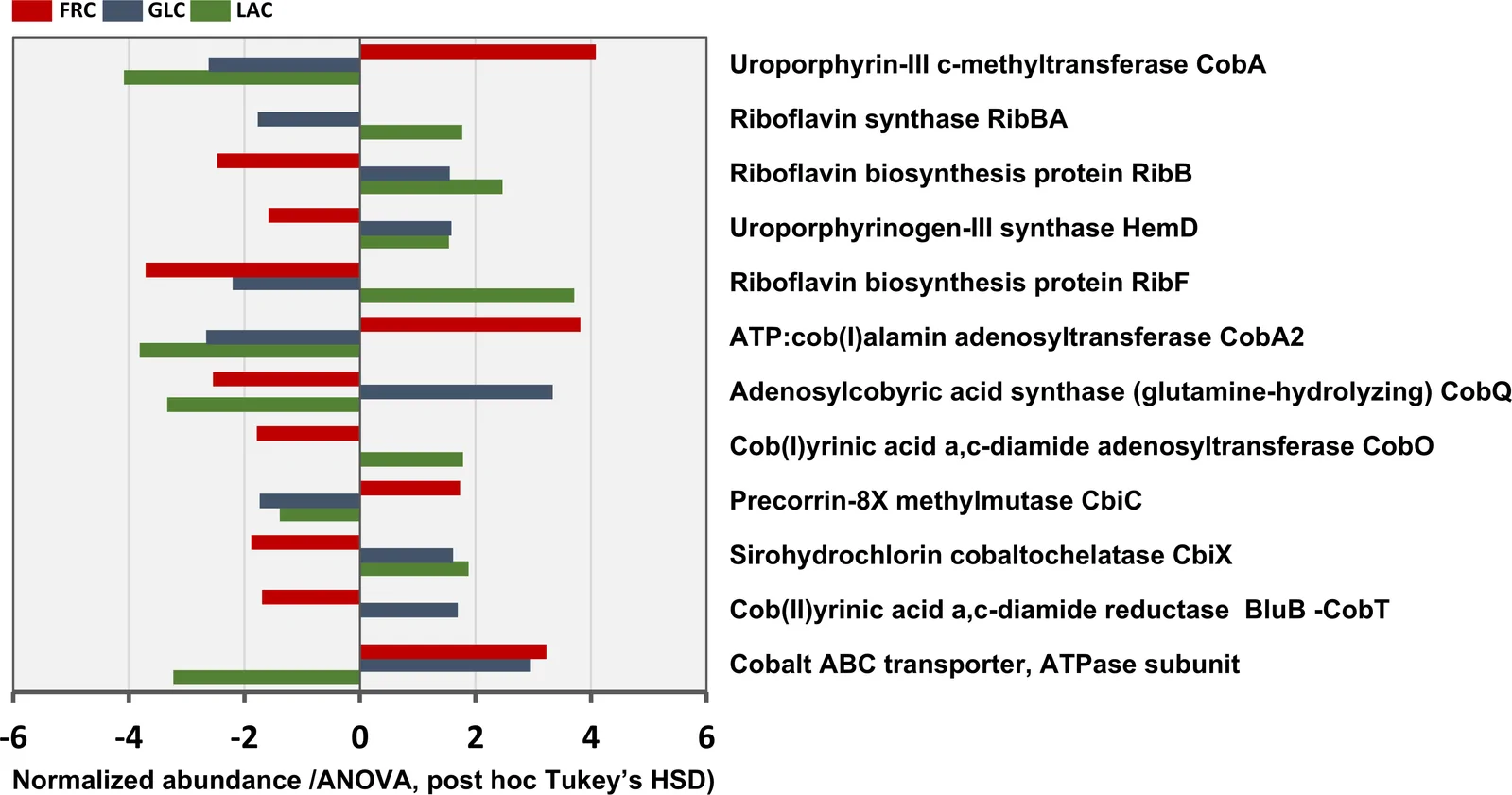
Carbon source-dependent abundance patterns of vitamin B12 biosynthesis, cobalt transport, and riboflavin pathway proteins. Proteins associated with *de novo* B12 biosynthesis, cobalt transport, and riboflavin metabolism showing significant abundance differences across lactate (LAC), glucose (GLC), and fructose (FRC) conditions. Values represent log₂-transformed abundance differences derived from proteome-wide ANOVA analysis. Early corrin ring assembly and adenosylation enzymes (e.g., CobA, CobA2, CobO) and the mid-pathway enzyme precorrin-8X methylmutase (CbiC) show carbon source-dependent variation, with several enriched under hexose conditions. In contrast, the late-stage amidation enzyme CobQ and early tetrapyrrole pathway enzymes (e.g., HemD, CbiX) display distinct patterns across substrates. Cobalt ABC transporter components are more abundant in hexose-grown cells, whereas riboflavin biosynthesis enzymes (RibBA, RibB, RibF) show higher abundance under lactate conditions. The terminal lower ligand synthesis enzyme (BluB-CobT) exhibits independent variation, indicating partial decoupling between precursor biosynthesis and final B12 assembly.

Proteomic analysis revealed distinct carbon source-dependent abundance patterns of proteins associated with vitamin B12 biosynthesis, cobalt transport, and riboflavin metabolism (Fig. 7; Table S2). Several enzymes involved in cobalamin biosynthesis showed differential enrichment across conditions, indicating carbon-specific regulation of B12 assembly pathways. Early-stage B12 biosynthesis enzymes, including CobA, CobA2, and the intermediate pathway enzyme CbiC, were most abundant under FRC conditions, consistent with the high volumetric B12 production observed during fructose metabolism. In contrast, the late-stage biosynthetic enzyme CobQ was most enriched in GLC-grown cells, whereas HemD and CbiX showed higher abundance in both GLC- and LAC-grown cultures relative to FRC, suggesting differential regulation of tetrapyrrole and cobalt-associated biosynthetic steps depending on the carbon source.

Proteins associated with cobalt uptake were similarly enriched under hexose conditions. Cobalt ABC transporter components displayed increased abundance in both GLC- and FRC-grown cells, supporting elevated trace metal demand during enhanced corrinoid biosynthesis under carbohydrate metabolism. In contrast, proteins involved in riboflavin biosynthesis and flavin metabolism were preferentially enriched during growth on lactate. RibBA showed the strongest response, with ~2.7-fold higher abundance in LAC relative to FRC cultures, whereas RibF and the riboflavin synthase alpha subunit RibB were elevated by ~1.7-fold and ~1.6-fold, respectively. These patterns are consistent with increased flavin cofactor generation and redox-associated metabolic activity during lactate metabolism, in agreement with the low residual riboflavin levels measured in LAC cultures.

Notably, the terminal B12 biosynthesis enzyme BluB-CobT2 displayed condition-specific regulation distinct from upstream pathway components, suggesting partial decoupling between precursor synthesis and final cobalamin assembly.

### Model of carbon source-dependent balancing between redox homeostasis and B12 biosynthesis in PFR

**Fig 8:**
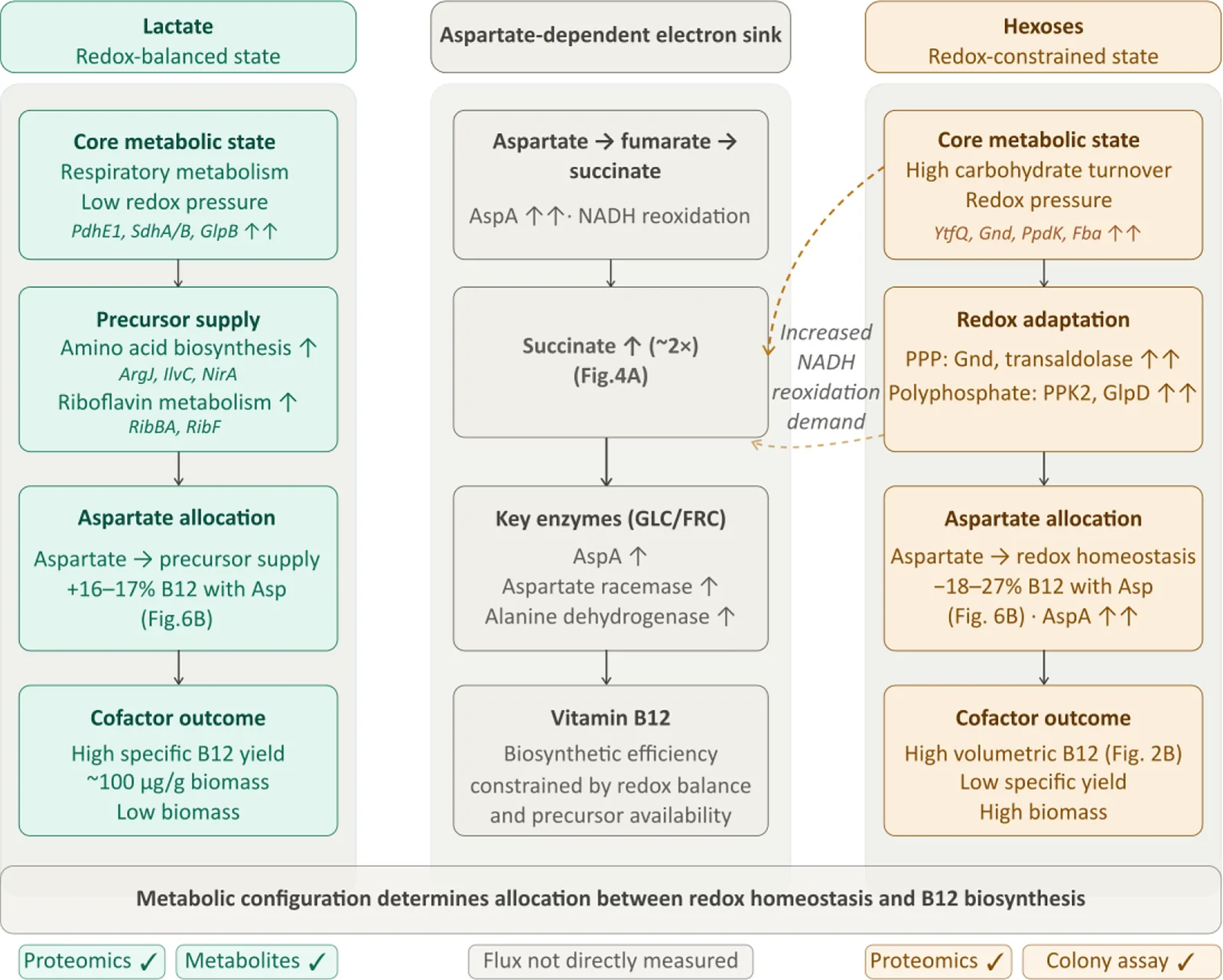
Model of carbon source-dependent metabolic configurations linking redox homeostasis and vitamin B12 biosynthesis in *P. freudenreichii*. Lactate (LAC) metabolism promotes a respiratory, comparatively redox-balanced configuration associated with efficient precursor supply, flavin metabolism, and high biomass-specific vitamin B12 production. In contrast, glucose and fructose (GLC/FRC) establish a redox-constrained configuration characterized by increased carbohydrate turnover, pentose phosphate pathway (PPP) activity, and redistribution of carbon and reducing power toward maintenance of redox homeostasis. A proposed feature of this configuration is activation of an aspartate-dependent electron sink, in which aspartate is converted to fumarate and subsequently reduced to succinate. Under specific conditions, fumarate may function as a terminal electron acceptor, redirecting carbon flux from propionate formation toward acetate and CO₂ production. Together, the model illustrates how carbon source influences carbon routing, resource allocation, and redox homeostasis, with vitamin B12 biosynthesis emerging as a system-level property of the prevailing metabolic configuration rather than biosynthetic enzyme abundance alone.

The concluding model (Fig. 8) was developed by integrating physiological, metabolite, amino acid, and LFQ proteomic data sets from LAC-, GLC-, and FRC-grown cells. Growth, metabolite, amino acid, vitamin, viability, and proteomic data sets were integrated to define carbon source-dependent physiological configurations. Collectively, our findings support a model in which PFR allocates metabolic resources between redox balancing and B12 biosynthesis according to carbon source. LAC promotes a respiratory, redox-balanced state associated with amino acid biosynthesis, flavin metabolism, and high biomass-specific B12 accumulation. In contrast, GLC and FRC induce a redox-constrained state characterized by increased carbohydrate turnover, pentose phosphate pathway activity, and succinate formation through the aspartate-to-fumarate pathway, which serves as an alternative electron sink. Under hexose conditions, aspartate is preferentially diverted toward redox balancing rather than corrinoid precursor supply, explaining its contrasting effects on B12 production in LAC- and FRC-grown cells. Thus, the prevailing physiological configuration determines the balance between redox homeostasis and efficient corrinoid biosynthesis.

## DISCUSSION

### Carbon source defines distinct physiological configurations

Here, we show that carbon source is a major determinant of metabolic organization in PFR, coordinating central carbon metabolism, redox homeostasis, amino acid utilization, and cofactor biosynthesis into distinct physiological states. It should be noted that carbon sources were supplied at equal mass concentrations (0.8% [wt/vol]) rather than equimolar or carbon-equivalent amounts; consequently, the observed quantitative differences in biomass yield and metabolite titers reflect both substrate identity and differences in the total available carbon per unit volume. Although this design limitation primarily affects quantitative comparisons of biomass and metabolite yields, it may also contribute to the magnitude of the physiological responses observed. Nevertheless, the coordinated pathway-specific proteomic patterns and the aspartate supplementation experiments support carbon source-dependent differences in metabolic organization. Integration of physiological, metabolite, amino acid, and proteomic data sets revealed that lactate and hexoses establish fundamentally different metabolic configurations that differentially allocate resources between respiratory metabolism, redox balancing, and vitamin B12 biosynthesis (Fig. 8). These observations are consistent with genome-scale modeling studies indicating that metabolic output in PFR is limited by the interplay between carbon flux distribution, energy generation, and redox balance, which together define feasible physiological states and pathway utilization (6). Combined with previous transcriptomic analyses showing extensive metabolic reprogramming during nutrient limitation and stationary-phase adaptation in *P. freudenreichii* (21, 39), our findings support a multi-layered regulatory framework in which carbon source and oxygen availability impose interconnected constraints on metabolism. Oxygen availability primarily influenced cofactor biosynthesis and stress adaptation, whereas carbon source defined the prevailing metabolic mode governing flux redistribution, redox handling, and biosynthetic prioritization.

A potential confound in comparative proteomic analyses across carbon sources with different growth rates is that proteome allocation varies with specific growth rate, although the direction and magnitude of these effects depend on the cultivation regime and the nature of the nutrient limitation (40, 41). Several features of our experimental design and data nevertheless support a substantial carbon source-dependent component. First, cells were harvested during mid-exponential growth in each condition, as established from growth curves obtained under the same cultivation conditions (Fig. S1), thereby minimizing proteome differences associated with growth-phase transitions. Second, proteomic responses were strongly pathway-specific and corresponded to the supplied substrates and associated physiological phenotypes. Lactate-grown cells were enriched in proteins involved in respiratory metabolism, Fe–S cluster assembly, sulfur assimilation, and riboflavin metabolism, whereas hexose-grown cells showed coordinated enrichment of carbohydrate transport, proteins involved in glycolytic and pentose phosphate pathways together with increased aspartate utilization and succinate formation. Taken together, these patterns support their interpretation primarily as carbon source-dependent metabolic configurations, while recognizing that differences in specific growth rate may also contribute to the observed proteome allocation. Definitive separation of substrate and growth-rate effects would require cultivation at controlled growth rates, for example in chemostats.

### Hexose metabolism establishes a redox-constrained carbohydrate-processing state

Hexose metabolism established a redox-constrained metabolic configuration characterized by increased carbohydrate turnover, pentose phosphate pathway (PPP) activity, overflow metabolism, and activation of alternative redox-balancing routes. Although specific growth rates were lower under hexose conditions than under lactate, the strong proteomic enrichment of glycolytic enzymes, carbohydrate transport systems, PPP components, phosphoketolase-related pathways, and polyphosphate-utilizing systems indicates that carbohydrate processing imposes a high demand for oxidizing equivalents relative to the cell’s respiratory capacity (42, 43). Increased abundance of polyphosphate kinase 2 (PPK2) and polyphosphate-glucose phosphotransferase further suggests enhanced turnover of intracellular polyphosphate. In *P. freudenreichii*, polyphosphate functions as a phosphate and energy storage polymer whose accumulation and utilization are strongly influenced by carbon source: lactate-grown cells accumulate large amounts of long-chain polyphosphate in volutin granules, whereas glucose-grown cells contain substantially lower levels, likely due to continuous utilization during carbohydrate phosphorylation (44). Although polyphosphate was not measured directly in the present study, the increased abundance of polyphosphate-associated proteins is consistent with enhanced phosphate and energy cycling accompanying rapid sugar-phosphate turnover under hexose metabolism.

In parallel, several observations point to increased reliance on an aspartate-dependent route for redox balancing. Hexose-grown cultures exhibited increased succinate production, strong induction of aspartate ammonia-lyase, rapid depletion of extracellular aspartate, and reduced aerobic colony-forming ability. Together, these findings support a model in which aspartate is converted to fumarate and subsequently reduced to succinate, providing an alternative electron sink under redox-constrained conditions. This interpretation is consistent with biochemical evidence linking aspartate deamination to fumarate reduction and succinate formation (45). Importantly, restoration of aerobic viability by aspartate supplementation provides experimental evidence supporting a role for aspartate-dependent redox balancing in survival during hexose metabolism.

### Lactate supports respiratory metabolism and efficient cellular B12 accumulation

In contrast, lactate promoted a respiratory and comparatively redox-balanced physiological state associated with amino acid biosynthesis, sulfur assimilation, Fe–S cluster assembly, and flavin-associated metabolism. Enrichment of respiratory proteins, including succinate dehydrogenase/fumarate reductase subunits, pyruvate dehydrogenase components, thioredoxin, and Fe–S assembly proteins, together with increased riboflavin pathway abundance and low residual riboflavin levels, indicates enhanced respiratory cofactor demand and active flavin metabolism during lactate growth. These observations are consistent with previous studies linking lactate metabolism and microaerobic respiration to efficient B12 production (2). Notably, lactate cultures displayed the highest biomass-specific B12 accumulation despite lower biomass formation, indicating that efficient corrinoid biosynthesis is associated with a balanced respiratory metabolic mode rather than maximal carbon throughput.

### Aspartate links redox balancing with B12 precursor allocation

Our data further demonstrate that carbon source-dependent metabolic adaptation strongly influences allocation of aspartate between redox balancing and corrinoid biosynthesis. Aspartate supplementation increased volumetric and specific B12 production during lactate metabolism but reduced B12 yields under fructose conditions. These opposing responses indicate that under lactate metabolism aspartate primarily supports biosynthetic precursor supply, whereas during hexose metabolism it is preferentially redirected toward succinate-forming pathways that support cellular redox homeostasis. Thus, metabolic competition between redox homeostasis and corrinoid precursor availability appears to be a key determinant of B12 biosynthetic efficiency.

### Corrinoid biosynthesis reflects metabolic state rather than enzyme abundance alone

The proteomic patterns observed for corrinoid biosynthesis further support this interpretation. Hexose metabolism, particularly fructose growth, enriched early-stage B12 biosynthesis enzymes and cobalt transport systems, consistent with high volumetric B12 production and elevated trace metal demand during carbohydrate-driven metabolism. In contrast, lactate preferentially enriched riboflavin biosynthesis and flavin-associated pathways, supporting respiratory metabolism and efficient biomass-specific corrinoid accumulation. The condition-specific regulation of the terminal enzyme BluB-CobT2 relative to upstream corrin ring biosynthesis proteins further suggests that final cobalamin assembly may be partially decoupled from precursor synthesis. Together, these observations indicate that B12 biosynthesis emerges from systems-level interactions among precursor availability, respiratory activity, redox balance, and carbon flux distribution rather than from biosynthetic enzyme abundance alone.

### Implications for metabolic state transitions and B12 production strategies

The integrated model proposed here (Fig. 8) describes two alternative metabolic configurations in PFR. Lactate supports a respiratory, flavin-associated, and biosynthetically efficient state favoring high cellular B12 accumulation, whereas glucose and fructose establish a redox-constrained physiological configuration characterized by elevated carbohydrate processing and enhanced pentose phosphate pathway activity. Because substrates were supplied at equal mass concentrations rather than carbon-equivalent concentrations, the observed differences reflect both substrate identity and the distinct carbon and redox inputs associated with each compound. These findings expand current understanding of metabolic program transitions in PFR and highlight how carbon source determines the balance between growth, redox adaptation, and cofactor biosynthesis. From an applied perspective, the results support two-stage cultivation strategies separating biomass production from optimized B12 synthesis, for example by combining rapid biomass generation under hexose-rich conditions with subsequent metabolic transition toward microaerobic, respiratory states favoring efficient corrinoid accumulation.

These findings also have implications for understanding PFR physiology under ecologically relevant conditions. In dairy fermentation, lactose is not directly utilizable by lactose-negative DSM 20271^T^, whereas lactate produced by lactic acid bacteria represents a major available carbon source (7). The present results suggest that PFR’s efficient respiratory metabolism on lactate is well matched to this ecological context, enabling high biomass-specific B12 accumulation from the predominant available substrate. In mixed-carbon environments such as cheese or the gastrointestinal tract, where PFR may encounter combinations of lactate, sugars, and amino acids simultaneously, the carbon source-dependent metabolic configurations characterized here likely operate in dynamic competition, with redox state serving as an integrating signal. Future studies using mixed-substrate or nutrient-shifting cultivation conditions would help establish how these physiological states are prioritized and regulated under ecologically realistic resource heterogeneity.

### Conclusions

This study demonstrates that carbon source is a major determinant of metabolic organization in *P. freudenreichii*, shaping how cells balance redox homeostasis, central carbon metabolism, and vitamin B12 biosynthesis. Integrated physiological, metabolite, amino acid, and proteomic analyses revealed that lactate and hexoses establish distinct metabolic configurations with different biosynthetic and redox-balancing priorities. Lactate supported a respiratory, comparatively redox-balanced state associated with amino acid biosynthesis, flavin metabolism, Fe–S cluster assembly, and efficient biomass-specific B12 accumulation. In contrast, glucose and fructose induced a redox-constrained state characterized by elevated carbohydrate processing, overflow metabolism, and activation of the aspartate–fumarate–succinate pathway as an alternative electron sink. The close association between aspartate metabolism, succinate formation, and aerobic viability suggests that aspartate-dependent redox balancing is an important adaptive mechanism during hexose metabolism. Our findings further indicate that B12 biosynthesis is regulated at the systems level rather than solely by biosynthetic enzyme abundance: carbon source-dependent differences in corrinoid biosynthesis proteins, cobalt transport, riboflavin metabolism, and aspartate utilization suggest that precursor allocation, respiratory activity, and redox homeostasis collectively determine corrinoid biosynthetic efficiency. Together, these findings establish a mechanistic framework linking carbon source-dependent metabolic configurations with redox homeostasis and B12 biosynthesis in *P. freudenreichii*, providing a basis for the rational optimization of industrial B12 production.

## Data Availability

The proteomics data sets generated during and/or analyzed during the current study are available in the PRIDE repository with data set identifier PXD079229. The numerical source data underlying the other results presented in this article are provided in the source data file in the supplemental material.

## SUPPLEMENTAL MATERIAL

Figure S1 msystems.01023-26-s0001.pdf Growth phase at the time of proteome harvest.

Figure S2 msystems.01023-26-s0002.pdf Venn diagrams comparing the number of all identified proteins within five biological replicate cells grown on fructose, glucose, and lactate.

Source Data msystems.01023-26-s0003.xlsx Numerical source data underlying Table 1 and the non-proteomic figures.

Table S1 msystems.01023-26-s0004.xlsx MaxOutput file listing of all identified proteins.

Table S2 msystems.01023-26-s0005.xlsx Differentially abundant proteins identified.

Table S3 msystems.01023-26-s0006.docx Selected proteins with significant abundance change in LAC, GLC, and FRC cells.

ASM does not own the copyrights to Supplemental Material that may be linked to, or accessed through, an article. The authors have granted ASM a non-exclusive, world-wide license to publish the Supplemental Material files. Please contact the corresponding author directly for reuse.

## References

[1] Thierry A, Deutsch SM, Falentin H, Dalmasso M, Cousin FJ, Jan G. 2011. New insights into physiology and metabolism of *Propionibacterium freudenreichii*. Int J Food Microbiol 149:19–27. doi:10.1016/j.ijfoodmicro.2011.04.026 21620505

[2] Dank A, Biel G, Abee T, Smid EJ. 2022. Microaerobic metabolism of lactate and propionate enhances vitamin B_12_ production in *Propionibacterium freudenreichii*. Microb Cell Fact 21:225. doi:10.1186/s12934-022-01945-8 PMC9617374 36307780

[3] Piveteau P. 1999. Metabolism of lactate and sugars by dairy propionibacteria: a review. Lait 79:23–41. doi:10.1051/lait:199912

[4] Orth JD, Thiele I, Palsson BØ. 2010. What is flux balance analysis? Nat Biotechnol 28:245–248. doi:10.1038/nbt.1614 PMC3108565 20212490

[5] Noor E, Flamholz A, Bar-Even A, Davidi D, Milo R, Liebermeister W. 2016. The protein cost of metabolic fluxes: prediction from enzymatic rate laws and cost minimization. PLoS Comput Biol 12:e1005167. doi:10.1371/journal.pcbi.1005167 PMC5094713 27812109

[6] Navone L, McCubbin T, Gonzalez-Garcia RA, Nielsen LK, Marcellin E. 2018. Genome-scale model guided design of *Propionibacterium* for enhanced propionic acid production. Metab Eng Commun 6:1–12. doi:10.1016/j.meteno.2017.11.001 PMC5725212 29255672

[7] Loux V, Mariadassou M, Almeida S, Chiapello H, Hammani A, Buratti J, Gendrault A, Barbe V, Aury JM, Deutsch SM, Parayre S, Madec MN, Chuat V, Jan G, Peterlongo P, Azevedo V, Le Loir Y, Falentin H. 2015. Mutations and genomic islands can explain the strain dependency of sugar utilization in 21 strains of *Propionibacterium freudenreichii*. BMC Genomics 16:296. doi:10.1186/s12864-015-1467-7 PMC4437456 25886522

[8] Deptula P, Laine PK, Roberts RJ, Smolander OP, Vihinen H, Piironen V, Paulin L, Jokitalo E, Savijoki K, Auvinen P, Varmanen P. 2017. *De novo* assembly of genomes from long sequence reads reveals uncharted territories of *Propionibacterium freudenreichii*. BMC Genomics 18:790. doi:10.1186/s12864-017-4165-9 PMC5644110 29037147

[9] Watanabe F, Yabuta Y, Bito T, Teng F. 2014. Vitamin B_12_-containing plant food sources for vegetarians. Nutrients 6:1861–1873. doi:10.3390/nu6051861 PMC4042564 24803097

[10] Temova Rakuša Ž, Roškar R, Hickey N, Geremia S. 2022. Vitamin B_12_ in foods, food supplements, and medicines—a review of its role and properties with a focus on its stability. Molecules 28:240. doi:10.3390/molecules28010240 PMC9822362 36615431

[11] Piwowarek K, Lipińska E, Hać-Szymańczuk E, Bzducha-Wróbel A, Synowiec A. 2018. Research on the ability of propionic acid and vitamin B_12_ biosynthesis by *Propionibacterium freudenreichii* strain T82. Antonie Van Leeuwenhoek 111:921–932. doi:10.1007/s10482-017-0991-7 PMC5945763 29178013

[12] Rodionov DA, Vitreschak AG, Mironov AA, Gelfand MS. 2003. Comparative genomics of the vitamin B_12_ metabolism and regulation in prokaryotes. J Biol Chem 278:41148–41159. doi:10.1074/jbc.M305837200 12869542

[13] Warren MJ, Raux E, Schubert HL, Escalante-Semerena JC. 2002. The biosynthesis of adenosylcobalamin (vitamin B_12_). Nat Prod Rep 19:390–412. doi:10.1039/b108967f 12195810

[14] Fang H, Kang J, Zhang D. 2017. Microbial production of vitamin B_12_: a review and future perspectives. Microb Cell Fact 16:15. doi:10.1186/s12934-017-0631-y PMC5282855 28137297

[15] Quesada-Chanto A, Schmid-Meyer AC, Schroeder AG, Carvalho-Jonas MF, Blanco I, Jonas R. 1998. Effect of oxygen supply on biomass, organic acids and vitamin B_12_ production by *Propionibacterium shermanii*. World J Microbiol Biotechnol 14:843–846. doi:10.1023/A:1008868907251

[16] Deptula P, Kylli P, Chamlagain B, Holm L, Kostiainen R, Piironen V, Savijoki K, Varmanen P. 2015. BluB/CobT2 fusion enzyme activity reveals mechanisms responsible for production of active form of vitamin B_12_ by *Propionibacterium freudenreichii*. Microb Cell Fact 14:186. doi:10.1186/s12934-015-0363-9 PMC4657239 26597297

[17] Zhang R, Sha Y, Chamlagain B, Edelmann M, Savijoki K, Piironen V, Deptula P, Varmanen P. 2025. Oxygen determines the requirement for cobalamin but not riboflavin in the growth of *Propionibacterium freudenreichii*. Sci Rep 15:27679. doi:10.1038/s41598-025-12983-8 PMC12307835 40731039

[18] Imlay JA. 2013. The molecular mechanisms and physiological consequences of oxidative stress: lessons from a model bacterium. Nat Rev Microbiol 11:443–454. doi:10.1038/nrmicro3032 PMC4018742 23712352

[19] Dank A, van Mastrigt O, Boeren S, Lillevang SK, Abee T, Smid EJ. 2021. *Propionibacterium freudenreichii* thrives in microaerobic conditions by complete oxidation of lactate to CO_2_. Environ Microbiol 23:3116–3129. doi:10.1111/1462-2920.15532 PMC8360058 33955639

[20] Falentin H, Deutsch SM, Jan G, Loux V, Thierry A, Parayre S, Maillard MB, Dherbécourt J, Cousin FJ, Jardin J, Siguier P, Couloux A, Barbe V, Vacherie B, Wincker P, Gibrat JF, Gaillardin C, Lortal S. 2010. The complete genome of *Propionibacterium freudenreichii* CIRM-BIA1^T^, a hardy actinobacterium with food and probiotic applications. PLoS One 5:e11748. doi:10.1371/journal.pone.0011748 PMC2909200 20668525

[21] Loivamaa I, Sillanpää A, Deptula P, Chamlagain B, Edelmann M, Auvinen P, Nyman TA, Savijoki K, Piironen V, Varmanen P. 2024. Aerobic adaptation and metabolic dynamics of *Propionibacterium freudenreichii* DSM 20271: insights from comparative transcriptomics and surfaceome analysis. mSystems 9:e00615-24. doi:10.1128/msystems.00615-24 PMC11494915 39345151

[22] Chamlagain B, Loivamaa I, Zhang R, Hovilehto E, Deptula P, Edelmann M, Kariluoto S, Piironen V, Varmanen P. 2026. Role of riboflavin and nicotinamide in vitamin B12 production by *Propionibacterium freudenreichii*. Int J Food Microbiol 444:111463. doi:10.1016/j.ijfoodmicro.2025.111463 40997606

[23] Fan Z, Chen Y, Zhang Y, Li Y, Wang P, Xiang Q, Yang R, Xie C. 2026. Cobalt availability modulate vitamin B12 biosynthesis in *Propionibacterium freudenreichii*: Insights from metabolic and transcriptional responses. Food Biosci 79:108747. doi:10.1016/j.fbio.2026.108747

[24] Chubukov V, Gerosa L, Kochanowski K, Sauer U. 2014. Coordination of microbial metabolism. Nat Rev Microbiol 12:327–340. doi:10.1038/nrmicro3238 24658329

[25] Koskinen P, Deptula P, Smolander OP, Tamene F, Kammonen J, Savijoki K, Paulin L, Piironen V, Auvinen P, Varmanen P. 2015. Complete genome sequence of *Propionibacterium freudenreichii* DSM 20271^T^. Stand Genomic Sci 10:83. doi:10.1186/s40793-015-0082-1 PMC4619572 26500719

[26] Begot C, Desnier I, Daudin JD, Labadie JC, Lebert A. 1996. Recommendations for calculating growth parameters by optical density measurements. J Microbiol Methods 25:225–232. doi:10.1016/0167-7012(95)00090-9

[27] Chamlagain B, Edelmann M, Kariluoto S, Ollilainen V, Piironen V. 2015. Ultra-high performance liquid chromatographic and mass spectrometric analysis of active vitamin B12 in cells of *Propionibacterium* and fermented cereal matrices. Food Chem 166:630–638. doi:10.1016/j.foodchem.2014.06.068 25053103

[28] Chamlagain B, Deptula P, Edelmann M, Kariluoto S, Grattepanche F, Lacroix C, Varmanen P, Piironen V. 2016. Effect of the lower ligand precursors on vitamin B12 production by food-grade propionibacteria. LWT Food Sci Technol 72:117–124. doi:10.1016/j.lwt.2016.04.023

[29] Xie C, Coda R, Chamlagain B, Edelmann M, Deptula P, Varmanen P, Piironen V, Katina K. 2018. *In situ* fortification of vitamin B12 in wheat flour and wheat bran by fermentation with *Propionibacterium freudenreichii*. J Cereal Sci 81:133–139. doi:10.1016/j.jcs.2018.05.002

[30] Kruk M, Varmanen P, Edelmann M, Chamlagain B, Trząskowska M. 2024. Food by-product valorisation in nutrients through spent brewer’s yeast bioprocessing with *Propionibacterium freudenreichii*. J Clean Prod 434:140102. doi:10.1016/j.jclepro.2023.140102

[31] Graça C, Edelmann M, Raymundo A, Sousa I, Coda R, Sontag-Strohm T, Huang X. 2022. Yoghurt as a starter in sourdough fermentation to improve the technological and functional properties of sourdough-wheat bread. J Funct Foods 88:104877. doi:10.1016/j.jff.2021.104877

[32] Foulston L, Elsholz AKW, DeFrancesco AS, Losick R. 2014. The extracellular matrix of *Staphylococcus aureus* biofilms comprises cytoplasmic proteins that associate with the cell surface in response to decreasing pH. mBio 5:e01667-14. doi:10.1128/mBio.01667-14 PMC4173787 25182325

[33] Batth TS, Tollenaere MX, Rüther P, Gonzalez-Franquesa A, Prabhakar BS, Bekker-Jensen S, Deshmukh AS, Olsen JV. 2019. Protein aggregation capture on microparticles enables multipurpose proteomics sample preparation. Mol Cell Proteomics 18:1027–1035. doi:10.1074/mcp.TIR118.001270 PMC6495262 30833379

[34] Cox J, Mann M. 2008. MaxQuant enables high peptide identification rates, individualized p.p.b.-range mass accuracies and proteome-wide protein quantification. Nat Biotechnol 26:1367–1372. doi:10.1038/nbt.1511 19029910

[35] Cox J, Neuhauser N, Michalski A, Scheltema RA, Olsen JV, Mann M. 2011. Andromeda: a peptide search engine integrated into the MaxQuant environment. J Proteome Res 10:1794–1805. doi:10.1021/pr101065j 21254760

[36] Deutsch EW, Csordas A, Sun Z, Jarnuczak A, Perez-Riverol Y, Ternent T, Campbell DS, Bernal-Llinares M, Okuda S, Kawano S, Moritz RL, Carver JJ, Wang M, Ishihama Y, Bandeira N, Hermjakob H, Vizcaíno JA. 2017. The ProteomeXchange consortium in 2017: supporting the cultural change in proteomics public data deposition. Nucleic Acids Res 45:D1100–D1106. doi:10.1093/nar/gkw936 PMC5210636 27924013

[37] Tyanova S, Temu T, Sinitcyn P, Carlson A, Hein MY, Geiger T, Mann M, Cox J. 2016. The Perseus computational platform for comprehensive analysis of (prote)omics data. Nat Methods 13:731–740. doi:10.1038/nmeth.3901 27348712

[38] Bateman A, Martin M-J, Orchard S, Magrane M, Ahmad S, Alpi E, Bowler-Barnett EH, Britto R, Bye-A-Jee H, Cukura A, et al.. 2023. UniProt: the Universal Protein Knowledgebase in 2023. Nucleic Acids Res 51:D523–D531. doi:10.1093/nar/gkac1052 PMC9825514 36408920

[39] Aburjaile FF, Rohmer M, Parrinello H, Maillard MB, Beaucher E, Henry G, Nicolas A, Madec MN, Thierry A, Parayre S, Deutsch SM, Cocaign-Bousquet M, Miyoshi A, Azevedo V, Le Loir Y, Falentin H. 2016. Adaptation of *Propionibacterium freudenreichii* to long-term survival under gradual nutritional shortage. BMC Genomics 17:1007. doi:10.1186/s12864-016-3367-x PMC5146858 27931189

[40] Hui S, Silverman JM, Chen SS, Erickson DW, Basan M, Wang J, Hwa T, Williamson JR. 2015. Quantitative proteomic analysis reveals a simple strategy of global resource allocation in bacteria. Mol Syst Biol 11:784. doi:10.15252/msb.20145697 PMC4358657 25678603

[41] Scott M, Gunderson CW, Mateescu EM, Zhang Z, Hwa T. 2010. Interdependence of cell growth and gene expression: origins and consequences. Science 330:1099–1102. doi:10.1126/science.1192588 21097934

[42] Kruger NJ, von Schaewen A. 2003. The oxidative pentose phosphate pathway: structure and organisation. Curr Opin Plant Biol 6:236–246. doi:10.1016/s1369-5266(03)00039-6 12753973

[43] Shimizu K, Matsuoka Y. 2019. Regulation of glycolytic flux and overflow metabolism depending on the source of energy generation for energy demand. Biotechnol Adv 37:284–305. doi:10.1016/j.biotechadv.2018.12.007 30576718

[44] Clark JE, Beegen H, Wood HG. 1986. Isolation of intact chains of polyphosphate from “*Propionibacterium shermanii*” grown on glucose or lactate. J Bacteriol 168:1212–1219. doi:10.1128/jb.168.3.1212-1219.1986 PMC213624 3782036

[45] Crow VL. 1986. Metabolism of aspartate by *Propionibacterium freudenreichii* subsp. *shermanii*: effect on lactate fermentation. Appl Environ Microbiol 52:359–365. doi:10.1128/aem.52.2.359-365.1986 PMC203530 16347135

