# Supplementary material for: Carbon source-dependent metabolic states govern redox homeostasis and cofactor biosynthesis in Propionibacterium freudenreichii
Source: mSystems. 2026 Sep 28;11(10):e01023-26. doi: 10.1128/msystems.01023-26 (PMC13712767; doi:10.1128/msystems.01023-26)
Supplement: Supplementary file 1 [file msystems.01023-26-s0001.pdf]

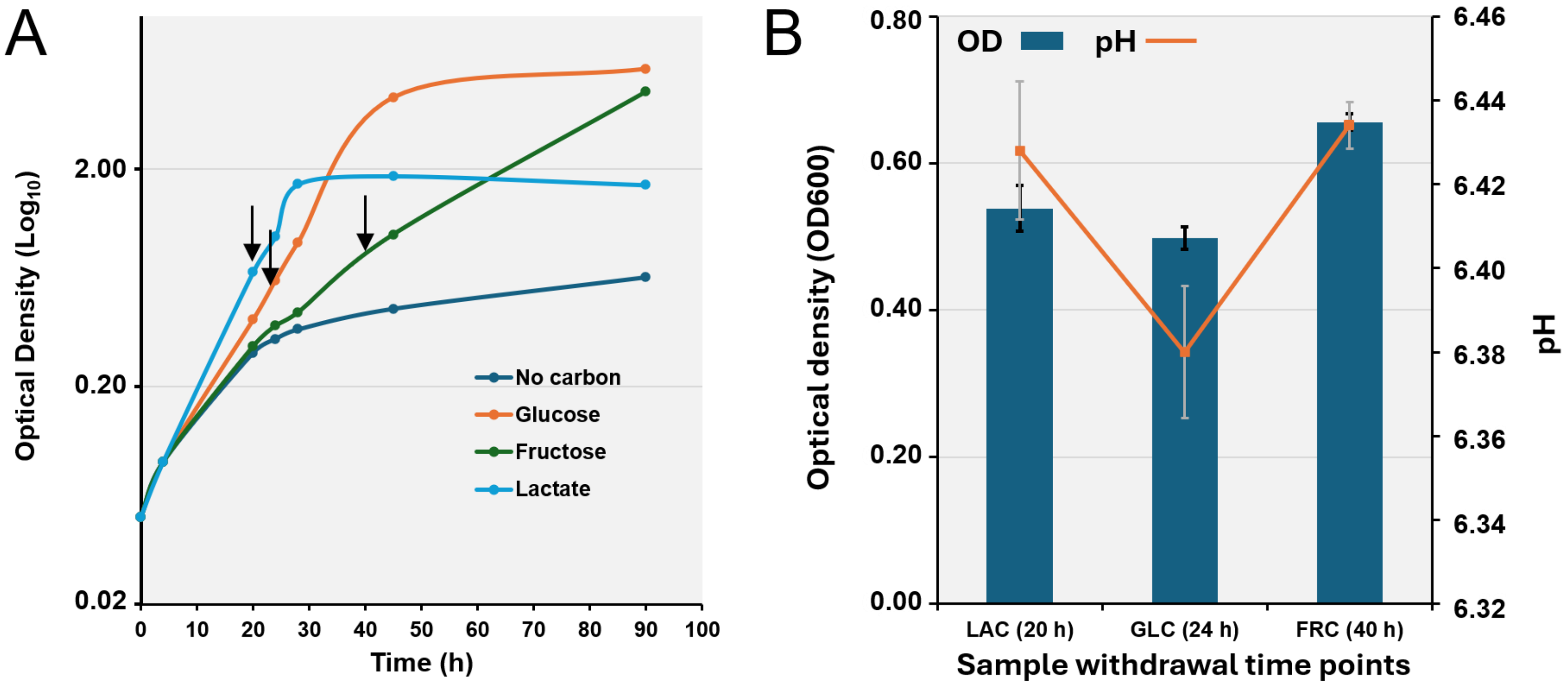

Figure S1. Growth phase at the time of proteome harvest. (A) Growth curves of *P. freudenreichii* DSM 20271^T in YE medium supplemented with lactate (LAC), glucose (GLC), or fructose (FRC) under the same flask conditions used for proteomics cultivations (20 mL anaerobic cultures at 30°C; two biological replicates per condition). Dashed vertical lines indicate the harvest time points for label-free quantitative proteomics (LAC: 20 h, GLC: 24 h, FRC: 40 h). (B) OD_{600} values of all five individual proteomics cultures at the time of harvest. Points represent individual biological replicates; bars indicate mean ± SD. OD_{600} values at harvest were 0.54 ± 0.03 (LAC), 0.50 ± 0.01 (GLC), and 0.66 ± 0.01 (FRC), confirming that all cultures were sampled during mid-exponential phase at comparable cell densities. Culture pH at harvest was 6.43 ± 0.02, 6.38 ± 0.02, and 6.43 ± 0.01 for LAC, GLC, and FRC respectively.
