# Supplementary material for: Carbon source-dependent metabolic states govern redox homeostasis and cofactor biosynthesis in Propionibacterium freudenreichii
Source: mSystems. 2026 Sep 28;11(10):e01023-26. doi: 10.1128/msystems.01023-26 (PMC13712767; doi:10.1128/msystems.01023-26)
Supplement: Supplementary file 2 [file msystems.01023-26-s0002.pdf]

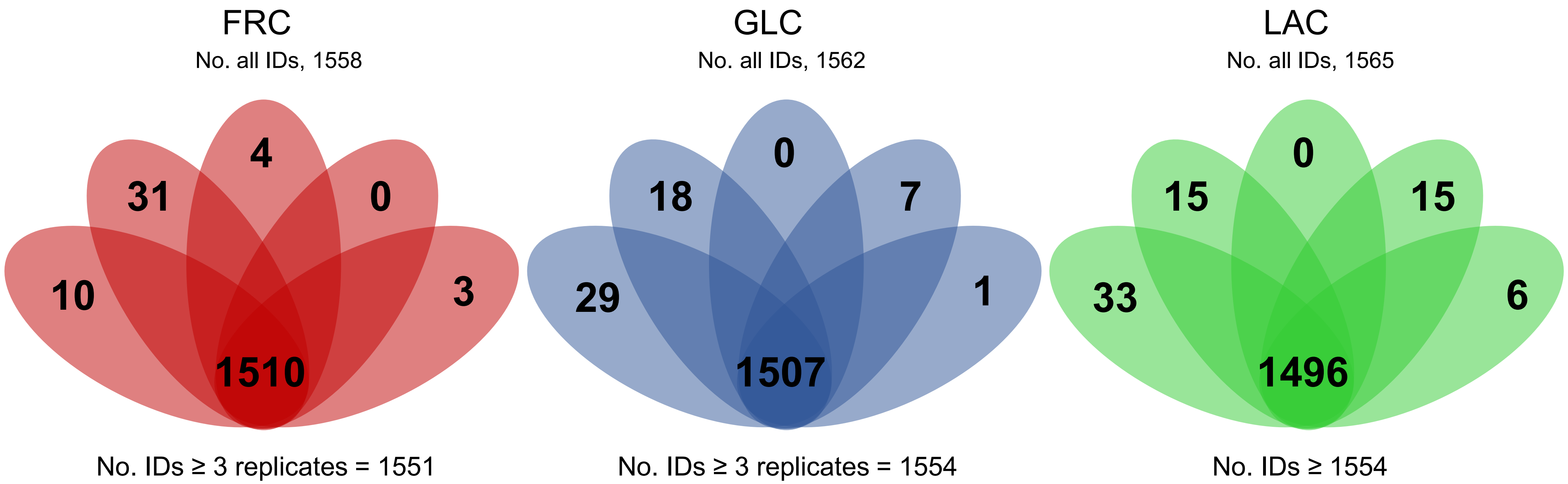

Figure S2. Venn diagrams comparing the number of all identified proteins within five biological replicate cells grown on fructose (FRC), glucose (GLC) and lactate (LAC). Proteome coverage was high across all three conditions; the number of uniquely detected proteins was low among the five biological replicates, and 96–99% of identified proteins were detected in at least three replicates.
