# Supplementary material for: Carbon source-dependent metabolic states govern redox homeostasis and cofactor biosynthesis in Propionibacterium freudenreichii
Source: mSystems. 2026 Sep 28;11(10):e01023-26. doi: 10.1128/msystems.01023-26 (PMC13712767; doi:10.1128/msystems.01023-26)
Supplement: Supplementary file 3 [file msystems.01023-26-s0006.docx]

**Table S3.** Selected proteins with significant abundance change in LAC (L), GLC (G) and FRC (F) cells.

| **PROTEIN ID** | **PROTEIN Name** | **Normalized abundance^(a^** | | |  |
| --- | --- | --- | --- | --- | --- |
| ***Proteins most abundant in LAC cells*** | | **LAC** | **GLC** | **FRC** | **Sign. Pairs** |
| AJQ90960.1_1224 | Succinate dehydrogenase/fumarate reductase Fe–S subunit | 8.9 | -8.9 | -8.5 | L_F;L_G |
| AJQ89939.1_203 | Dihydrolipoamide acyltransferase | 8.1 | -7.9 | -8.1 | L_F;L_G |
| AJQ91184.1_1448 | Penicillin binding protein transpeptidase domain protein | 7.6 | -7.6 | -7.4 | L_F;L_G |
| AJQ90619.1_883 | Anaerobic glycerol-3-phosphate dehydrogenase subunit B | 7.2 | -7.2 | -5.2 | F_G;L_G;L_F |
| AJQ90991.1_1255 | Ketol-acid reductoisomerase | 7.2 | -6.3 | -7.2 | G_F;L_F;L_G |
| AJQ90961.1_1225 | Succinate dehydrogenase flavoprotein subunit | 6.9 | -6.9 | -6.9 | L_F;L_G |
| AJQ89937.1_201 | Pyruvate dehydrogenase E1 component, alpha subunit | 6.6 | -5.9 | -6.6 | L_F;L_G |
| AJQ91736.1_2000 | Ferredoxin-nitrite reductase | 6.5 | -5.0 | -6.5 | G_F;L_F;L_G |
| AJQ91031.1_1295 | Arginine biosynthesis bifunctional protein ArgJ | 6.5 | -6.1 | -6.5 | L_F;L_G |
| AJQ90030.1_294 | NLPA lipoprotein | 6.3 | -5.2 | -6.3 | G_F;L_F;L_G |
| AJQ91127.1_1391 | FeS assembly protein SufD | 6.2 | -5.5 | -6.2 | L_F;L_G |
| AJQ89889.1_153 | Argininosuccinate synthase | 6.1 | -4.7 | -6.1 | G_F;L_F;L_G |
| AJQ90929.1_1193 | Peptide chain release factor 2 | 6.1 | -5.3 | -6.1 | L_F;L_G |
| AJQ90942.1_1206 | Malonyl-CoA-acyl carrier protein transacylase | 6.0 | -3.7 | -6.0 | G_F;L_F;L_G |
| AJQ90968.1_1232 | Metal transport system ABC transporter substrate-binding | 5.8 | -4.3 | -5.8 | G_F;L_F;L_G |
| AJQ90604.1_868 | RecA protein | 5.8 | -4.6 | -5.8 | G_F;L_F;L_G |
| AJQ89898.1_162 | 2,5-diketo-D-gluconic acid reductase A | 5.8 | -5.8 | -5.4 | L_F;L_G |
| AJQ91580.1_1844 | Ferrous iron transport protein A | 5.6 | -4.0 | -5.6 | G_F;L_F;L_G |
| AJQ89842.1_106 | Arginine/ornithine binding protein | 5.6 | -5.2 | -5.6 | L_F;L_G |
| AJQ91432.1_1696 | Type I antifreeze protein | 5.6 | -3.0 | -5.6 | G_F;L_F;L_G |
| AJQ91067.1_1331 | 30S ribosomal protein S16 | 5.5 | -5.5 | -2.7 | F_G;L_G;L_F |
| AJQ91569.1_1833 | Thioredoxin | 5.4 | -3.3 | -5.4 | G_F;L_F;L_G |
| AJQ91688.1_1952 | Replicative DNA helicase | 5.4 | 3.7 | -5.4 | G_F;L_F;L_G |
| AJQ90330.1_594 | Osmoprotectant transport ATP-binding protein | 5.4 | -5.1 | -5.4 | L_F;L_G |
| AJQ90653.1_917 | Putative glutathione S-transferase | 5.4 | -5.4 | -4.6 | L_G;L_F |
| AJQ91479.1_1743 | FCD domain protein | 5.3 | -3.3 | -5.3 | G_F;L_F;L_G |
| AJQ90962.1_1226 | Succinate dehydrogenase cytochrome B subunit, b558 | 5.2 | -5.2 | -4.7 | L_G;L_F |
| AJQ91264.1_1528 | Phosphinothricin acetyltransferase | 5.2 | -5.2 | -4.6 | L_G;L_F |
| AJQ91593.1_1857 | GTP-binding translation elongation factor typA | 5.2 | -3.6 | -5.2 | G_F;L_F;L_G |
| AJQ90945.1_1209 | 3-oxoacyl-[acyl-carrier-protein] synthase II | 5.2 | -3.5 | -5.2 | G_F;L_F;L_G |
| AJQ92027.1_2291 | YceI family protein | 5.1 | -5.1 | -4.0 | L_G;L_F |
| AJQ90308.1_572 | TRNA (guanine-N(7)-)-methyltransferase | 5.1 | -3.6 | -5.1 | G_F;L_F;L_G |
| AJQ91594.1_1858 | Folate-binding protein YgfZ | 5.1 | -3.7 | -5.1 | G_F;L_F;L_G |
| AJQ90590.1_854 | 3-isopropylmalate dehydratase small subunit | 5.1 | -5.0 | -5.1 | L_F;L_G |
| AJQ91739.1_2003 | Sulfate adenylyltransferase subunit 1 | 5.0 | 2.7 | -5.0 | G_F;L_F;L_G |
| AJQ90366.1_630 | Transposase InsI for insertion sequence element IS30C | 5.0 | -4.8 | -5.0 | L_F;L_G |
| AJQ91100.1_1364 | Proline-tRNA ligase | 5.0 | -4.1 | -5.0 | L_F;L_G |
| AJQ91738.1_2002 | Sulfate adenylyltransferase subunit 2 | 5.0 | -2.6 | -5.0 | G_F;L_F;L_G |
| AJQ91478.1_1742 | L-lactate permease | 5.0 | -5.0 | -4.7 | L_F;L_G |
| AJQ91461.1_1725 | 60 kDa chaperonin 1 | 4.9 | -3.8 | -4.9 | L_F;L_G |
| AJQ91493.1_1757 | Co-factor-dependent phosphoglycerate mutase | 4.9 | -4.9 | 2.6 | F_G;L_G;L_F |
| AJQ89885.1_149 | Putative periplasmic or exported protein | 4.8 | -2.7 | -4.8 | G_F;L_F;L_G |
| AJQ91634.1_1898 | ATP-dependent protease ATP-binding subunit clpC1 | 4.8 | -4.0 | -4.8 | L_F;L_G |
| AJQ91221.1_1485 | Macrolide-transport ATP-binding protein ABC transporter | 4.8 | -4.7 | -4.8 | L_F;L_G |
| AJQ90260.1_524 | 50S ribosomal protein L23 | 4.7 | -4.4 | -4.7 | L_F;L_G |
| AJQ90611.1_875 | GTPase HflX | 4.6 | -3.2 | -4.6 | G_F;L_F;L_G |
| AJQ90585.1_849 | 2-isopropylmalate synthase/homocitrate synthase | 4.5 | -3.5 | -4.5 | L_F;L_G |
| AJQ90272.1_536 | 30S ribosomal protein S8 | 4.5 | -4.3 | -4.5 | L_F;L_G |
| AJQ91029.1_1293 | Acetylornithine transaminase | 4.5 | -3.4 | -4.5 | L_F;L_G |
| AJQ91176.1_1440 | POTRA domain protein, FtsQ-type | 4.5 | -4.5 | -2.9 | L_G;L_F |
| AJQ90710.1_974 | ABC-type uncharacterized transport system | 4.5 | -3.7 | -4.5 | L_F;L_G |
| AJQ90232.1_496 | Alkyl hydroperoxide reductase, F subunit | 4.4 | 2.2 | -4.4 | G_F;L_F;L_G |
| AJQ91933.1_2197 | Osmoprotectant proV | 4.4 | -4.4 | 3.5 | F_G;L_G |
| AJQ89777.1_41 | Putative hippurate hydrolase | 4.4 | -2.7 | -4.4 | G_F;L_F;L_G |
| AJQ90964.1_1228 | Quinolinate synthase A | 4.4 | 2.3 | -4.4 | G_F;L_F;L_G |
| AJQ90479.1_743 | Oligoribonuclease | 4.4 | -4.4 | -4.1 | L_F;L_G |
| AJQ91270.1_1534 | Cysteine synthase | 4.4 | 2.7 | -4.4 | G_F;L_F;L_G |
| AJQ89938.1_202 | Branched-chain alpha-keto acid dehydrogenase E1 beta | 4.4 | -2.9 | -4.4 | G_F;L_F;L_G |
| AJQ90470.1_734 | Putative L-ribulose-5-phosphate 4-epimerase | 4.4 | -2.3 | -4.4 | G_F;L_F;L_G |
| AJQ91030.1_1294 | Acetylglutamate kinase | 4.4 | -3.7 | -4.4 | L_F;L_G |
| AJQ89908.1_172 | Panthothenate kinase | 4.4 | -3.7 | -4.4 | L_F;L_G |
| AJQ90031.1_295 | Methionine import ATP-binding protein MetN 2 | 4.4 | -2.9 | -4.4 | G_F;L_F;L_G |
| AJQ91737.1_2001 | Phosphoadenosine phosphosulfate reductase | 4.4 | 2.5 | -4.4 | G_F;L_F;L_G |
| AJQ90583.1_847 | 3-isopropylmalate dehydrogenase | 4.3 | -4.3 | -3.9 | L_G;L_F |
| AJQ90162.1_426 | Chaperone protein DnaK | 4.3 | -3.6 | -4.3 | L_F;L_G |
| AJQ91363.1_1627 | Aminoacyl-tRNA hydrolase | 4.3 | -4.3 | -3.9 | L_F;L_G |
| AJQ90334.1_598 | 60 kDa chaperonin 1 | 4.3 | -2.1 | -4.3 | G_F;L_F;L_G |
| AJQ91319.1_1583 | Putative flagellar protein FliS | 4.3 | -4.3 | 2.2 | F_G;L_G |
| AJQ90531.1_795 | GTPase obg | 4.3 | -3.1 | -4.3 | L_F;L_G |
| AJQ91266.1_1530 | Hippurate hydrolase | 4.3 | -4.3 | 3.3 | F_G;L_G |
| AJQ90809.1_1073 | High-affinity BCA transport ATP-binding protein livF | 4.2 | -4.2 | -3.8 | L_G;L_F |
| AJQ91400.1_1664 | Heat shock protein transcriptional repressor HspR1 | 4.2 | -4.2 | -3.4 | L_G;L_F |
| AJQ89737.1_1 | Chromosomal replication initiator protein DnaA | 4.2 | -2.4 | -4.2 | G_F;L_F;L_G |
| AJQ91488.1_1752 | Transcriptional regulator, CarD family | 4.2 | -2.2 | -4.2 | G_F;L_F;L_G |
| AJQ90511.1_775 | ATP-dependent Clp protease ATP-binding subunit ClpX | 4.2 | 2.4 | -4.2 | G_F;L_F;L_G |
| AJQ90527.1_791 | Radical SAM domain protein | 4.1 | -4.1 | -3.1 | L_G;L_F |
| AJQ90712.1_976 | ABC transporter substrate binding protein | 4.1 | -4.1 | -2.0 | L_G;L_F |
| AJQ91124.1_1388 | Cysteine desulfurase, SufS family protein | 4.1 | -2.7 | -4.1 | L_F;L_G |
| AJQ90557.1_821 | Heat-inducible transcription repressor hrcA | 4.1 | -2.3 | -4.1 | G_F;L_F;L_G |
| AJQ90023.1_287 | Mrr restriction system protein | 4.1 | 2.7 | -4.1 | G_F;L_F |
| AJQ91384.1_1648 | Glutamate dehydrogenase | 4.1 | -2.3 | -4.1 | G_F;L_F;L_G |
| AJQ90487.1_751 | ATP-binding cassette protein, ChvD family | 4.1 | -2.0 | -4.1 | G_F;L_F;L_G |
| AJQ91125.1_1389 | FeS assembly ATPase SufC | 4.1 | -3.8 | -4.1 | L_F;L_G |
| AJQ90295.1_559 | DNA-directed RNA polymerase, alpha subunit | 4.0 | -2.3 | -4.0 | G_F;L_F;L_G |
| AJQ91151.1_1415 | Two-component system response regulator | 4.0 | 2.0 | -4.0 | G_F;L_F;L_G |
| AJQ90630.1_894 | RNA polymerase sigma factor | 4.0 | 2.0 | -4.0 | G_F;L_F;L_G |
| AJQ90857.1_1121 | Peptide chain release factor 1 | 4.0 | -2.7 | -4.0 | L_F;L_G |
| AJQ91476.1_1740 | Iron-sulfur cluster-binding protein | 4.0 | -4.0 | -3.6 | L_F;L_G |
| ***Proteins most abundant in GLC cells*** | | **LAC** | **GLC** | **FRC** | **Sign. Pairs** |
| AJQ91599.1_1863 | Inositol-3-phosphate synthase | -11.7 | 11.7 | 11.4 | G_L;F_L |
| AJQ90350.1_614 | ABC transporter periplasmic-binding protein ytfQ | -11.2 | 11.1 | 11.2 | G_L;F_L;F_G |
| AJQ90025.1_289 | Pyruvate, phosphate dikinase | -10.9 | 10.9 | 10.4 | G_L;F_L |
| AJQ90161.1_425 | Phosphogluconate dehydrogenase (decarboxylating) | -10.8 | 10.5 | 10.8 | G_L;F_L;F_G |
| AJQ91378.1_1642 | Galactokinase | -8.9 | 8.9 | 8.8 | G_L;F_L |
| AJQ91259.1_1523 | Aspartate ammonia-lyase | -9.5 | 8.7 | 9.5 | G_L;F_L;F_G |
| AJQ91818.1_2082 | Aldose 1-epimerase | -7.9 | 7.9 | 7.8 | G_L;F_L |
| AJQ90310.1_574 | Elongation factor G fusA2 | -8.7 | 7.9 | 8.7 | G_L;F_L;F_G |
| AJQ91900.1_2164 | Glycerol-3-phosphate dehydrogenase | -7.7 | 7.7 | 7.6 | G_L;F_L |
| AJQ91831.1_2095 | DAK1 domain protein | -7.6 | 7.1 | 7.6 | G_L;F_L;F_G |
| AJQ89849.1_113 | 4-hydroxythreonine-4-phosphate dehydrogenase 2 | -6.9 | 6.9 | 6.6 | G_L;F_L |
| AJQ91822.1_2086 | Ribulokinase | -6.8 | 6.8 | 5.2 | F_L;G_L |
| AJQ91515.1_1779 | Inositol 2-dehydrogenase | -6.8 | 6.8 | 4.2 | F_L;G_L;G_F |
| AJQ90866.1_1130 | 2-oxoglutarate decarboxylase | -6.8 | 6.8 | 6.4 | G_L;F_L |
| AJQ91518.1_1782 | Putative 5-dehydro-2-deoxygluconokinase | -6.5 | 6.5 | 4.4 | F_L;G_L;G_F |
| AJQ90376.1_640 | Isocitrate dehydrogenase [NADP] | -6.3 | 6.3 | 6.0 | G_L;F_L |
| AJQ91279.1_1543 | Carbon starvation protein A | -6.2 | 6.2 | 6.2 | G_L;F_L |
| AJQ91278.1_1542 | Glycerate kinase | -7.0 | 6.2 | 7.0 | G_L;F_L;F_G |
| AJQ91517.1_1781 | UbiC transcription regulator-associated | -6.1 | 6.1 | 5.6 | F_L;G_L |
| AJQ91560.1_1824 | Phosphoenolpyruvate-protein phosphotransferase | -7.2 | 5.9 | 7.2 | G_L;F_L;F_G |
| AJQ90373.1_637 | Bifunctional protein FolD | -5.7 | 5.7 | 3.3 | F_L;G_L;G_F |
| AJQ91519.1_1783 | Deoxyribose-phosphate aldolase | -5.5 | 5.5 | 3.7 | F_L;G_L |
| AJQ91758.1_2022 | ThiS family protein | -5.5 | 5.5 | 4.9 | F_L;G_L |
| AJQ91261.1_1525 | Aspartate racemase | -6.7 | 5.4 | 6.7 | G_L;F_L;F_G |
| AJQ91255.1_1519 | Glyoxylate reductase | -5.4 | 5.4 | 5.3 | G_L;F_L |
| AJQ89780.1_44 | Alanine dehydrogenase | -7.0 | 5.3 | 7.0 | G_L;F_L;F_G |
| AJQ90170.1_434 | Formate-tetrahydrofolate ligase | -4.8 | 4.8 | 4.1 | F_L;G_L |
| AJQ90132.1_396 | Acyl-CoA dehydrogenase, short-chain specific | -5.8 | 4.7 | 5.8 | G_L;F_L;F_G |
| AJQ91003.1_1267 | Protein PafC | -6.2 | 4.6 | 6.2 | G_L;F_L;F_G |
| AJQ91824.1_2088 | FGGY-family pentulose kinase | -4.6 | 4.6 | 2.0 | F_L;G_L;G_F |
| AJQ91521.1_1785 | 3D-(3,5/4)-trihydroxycyclohexane-1,2-dione hydrolase | -4.6 | 4.6 | 2.3 | F_L;G_L;G_F |
| AJQ91237.1_1501 | Citrate lyase beta subunit | -4.5 | 4.5 | 2.8 | F_L;G_L |
| AJQ90606.1_870 | Ribonuclease Y | -4.8 | 4.5 | 4.8 | G_L;F_L |
| AJQ90488.1_752 | Dihydroxyacetone kinase, DhaK subunit | -5.4 | 4.5 | 5.4 | G_L;F_L |
| AJQ91606.1_1870 | Putative HYDROLASE MUTT1 | -6.0 | 4.4 | 6.0 | G_L;F_L;F_G |
| AJQ91516.1_1780 | Transaldolase | -4.4 | 4.4 | 2.0 | F_L;G_L;G_F |
| AJQ90889.1_1153 | Prolyl aminopeptidase | -6.6 | 4.4 | 6.6 | G_L;F_L;F_G |
| AJQ90663.1_927 | Polyphosphate:nucleotide phosphotransferase, PPK2 | -5.9 | 4.4 | 5.9 | G_L;F_L;F_G |
| ***Proteins most abundant in FRC cells*** | | **LAC** | **GLC** | **FRC** | **Sign. Pairs** |
| AJQ91599.1_1863 | Inositol-3-phosphate synthase | -11.7 | 11.7 | 11.4 | G_L;F_L |
| AJQ90350.1_614 | ABC transporter periplasmic-binding protein ytfQ | -11.2 | 11.1 | 11.2 | G_L;F_L;F_G |
| AJQ90161.1_425 | Phosphogluconate dehydrogenase (decarboxylating) | -10.8 | 10.5 | 10.8 | G_L;F_L;F_G |
| AJQ90025.1_289 | Pyruvate, phosphate dikinase | -10.9 | 10.9 | 10.4 | G_L;F_L |
| AJQ91259.1_1523 | Aspartate ammonia-lyase | -9.5 | 8.7 | 9.5 | G_L;F_L;F_G |
| AJQ91378.1_1642 | Galactokinase | -8.9 | 8.9 | 8.8 | G_L;F_L |
| AJQ90310.1_574 | Elongation factor G fusA2 | -8.7 | 7.9 | 8.7 | G_L;F_L;F_G |
| AJQ91818.1_2082 | Aldose 1-epimerase | -7.9 | 7.9 | 7.8 | G_L;F_L |
| AJQ91900.1_2164 | Glycerol-3-phosphate dehydrogenase | -7.7 | 7.7 | 7.6 | G_L;F_L |
| AJQ91560.1_1824 | Phosphoenolpyruvate-protein phosphotransferase | -7.2 | 5.9 | 7.2 | G_L;F_L;F_G |
| AJQ89780.1_44 | Alanine dehydrogenase | -7.0 | 5.3 | 7.0 | G_L;F_L;F_G |
| AJQ91278.1_1542 | Glycerate kinase | -7.0 | 6.2 | 7.0 | G_L;F_L;F_G |
| AJQ91261.1_1525 | Aspartate racemase | -6.7 | 5.4 | 6.7 | G_L;F_L;F_G |
| AJQ90889.1_1153 | Prolyl aminopeptidase | -6.6 | 4.4 | 6.6 | G_L;F_L;F_G |
| AJQ89849.1_113 | 4-hydroxythreonine-4-phosphate dehydrogenase 2 | -6.9 | 6.9 | 6.6 | G_L;F_L |
| AJQ90866.1_1130 | 2-oxoglutarate decarboxylase | -6.8 | 6.8 | 6.4 | G_L;F_L |
| AJQ91091.1_1355 | Glycerol kinase | -6.3 | 3.7 | 6.3 | G_L;F_L;F_G |
| AJQ89940.1_204 | UDP-N-acetylglucosamine diphosphorylase | -6.2 | 4.0 | 6.2 | G_L;F_L;F_G |
| AJQ91003.1_1267 | Protein PafC | -6.2 | 4.6 | 6.2 | G_L;F_L;F_G |
| AJQ91279.1_1543 | Carbon starvation protein A | -6.2 | 6.2 | 6.2 | G_L;F_L |
| AJQ90623.1_887 | putative phosphoketolase | -6.1 | 4.4 | 6.1 | G_L;F_L;F_G |
| AJQ91606.1_1870 | Putative HYDROLASE MUTT1 | -6.0 | 4.4 | 6.0 | G_L;F_L;F_G |
| AJQ90376.1_640 | Isocitrate dehydrogenase [NADP] | -6.3 | 6.3 | 6.0 | G_L;F_L |
| AJQ90663.1_927 | Polyphosphate:nucleotide phosphotransferase, PPK2 | -5.9 | 4.4 | 5.9 | G_L;F_L;F_G |
| AJQ90489.1_753 | Dihydroxyacetone kinase, L subunit | -5.9 | 3.9 | 5.9 | G_L;F_L;F_G |
| AJQ90132.1_396 | Acyl-CoA dehydrogenase, short-chain specific | -5.8 | 4.7 | 5.8 | G_L;F_L;F_G |
| AJQ92010.1_2274 | Cytochrome P450 | -5.8 | 3.4 | 5.8 | G_L;F_L;F_G |
| AJQ91517.1_1781 | UbiC transcription regulator-associated | -6.1 | 6.1 | 5.6 | F_L;G_L |
| AJQ91542.1_1806 | Putative aldehyde dehydrogenase AldA | -5.6 | 3.1 | 5.6 | G_L;F_L;F_G |
| AJQ91470.1_1734 | L-serine dehydratase 1 | -5.6 | 4.0 | 5.6 | G_L;F_L;F_G |
| AJQ90833.1_1097 | Phosphoglucomutase | -5.5 | 3.5 | 5.5 | G_L;F_L;F_G |
| AJQ89881.1_145 | PTS system mannose-specific EIIBCA component | -5.5 | 3.3 | 5.5 | G_L;F_L;F_G |
| AJQ90831.1_1095 | 1,4-alpha-glucan branching enzyme GlgB | -5.5 | 4.1 | 5.5 | G_L;F_L;F_G |
| AJQ90128.1_392 | Glucose-6-phosphate isomerase | -5.5 | 3.5 | 5.5 | G_L;F_L;F_G |
| AJQ90488.1_752 | Dihydroxyacetone kinase, DhaK subunit | -5.4 | 4.5 | 5.4 | G_L;F_L |
| AJQ89865.1_129 | Gluconokinase | -5.4 | 4.3 | 5.4 | G_L;F_L;F_G |
| AJQ89976.1_240 | Polyphosphate-glucose phosphotransferase | -5.4 | -3.0 | 5.4 | G_L;F_L;F_G |
| AJQ91255.1_1519 | Glyoxylate reductase | -5.4 | 5.4 | 5.3 | G_L;F_L |
| AJQ91822.1_2086 | Ribulokinase | -6.8 | 6.8 | 5.2 | F_L;G_L |
| AJQ90498.1_762 | Phosphogluconate dehydrogenase (decarboxylating) | -5.0 | 3.3 | 5.0 | G_L;F_L;F_G |
| AJQ91068.1_1332 | M38 family peptidase | -4.9 | -2.8 | 4.9 | G_L;F_L;F_G |
| AJQ91758.1_2022 | ThiS family protein | -5.5 | 5.5 | 4.9 | F_L;G_L |
| AJQ91245.1_1509 | Starch synthase | -4.8 | 3.3 | 4.8 | G_L;F_L;F_G |
| AJQ90606.1_870 | Ribonuclease Y | -4.8 | 4.5 | 4.8 | G_L;F_L |
| AJQ90115.1_379 | Prophage Lp2 protein 6 | -4.5 | -2.3 | 4.5 | G_L;F_L;F_G |
| AJQ91518.1_1782 | Putative 5-dehydro-2-deoxygluconokinase | -6.5 | 6.5 | 4.4 | F_L;G_L;G_F |
| AJQ90812.1_1076 | Dipeptidase pepE | -4.3 | -3.1 | 4.3 | F_L;F_G |
| AJQ91244.1_1508 | Glucose-1-phosphate adenylyltransferase | -4.2 | -2.9 | 4.2 | F_L;F_G |
| AJQ91515.1_1779 | Inositol 2-dehydrogenase | -6.8 | 6.8 | 4.2 | F_L;G_L;G_F |
| AJQ90012.1_276 | Succinate CoA transferase | -4.2 | 2.4 | 4.2 | G_L;F_L;F_G |
| AJQ90818.1_1082 | Amino acid-binding ACT domain protein | -4.1 | 2.5 | 4.1 | G_L;F_L;F_G |
| AJQ90170.1_434 | Formate-tetrahydrofolate ligase | -4.8 | 4.8 | 4.1 | F_L;G_L |
| AJQ92026.1_2290 | Fructose-bisphosphate aldolase class 1 | -4.1 | -2.7 | 4.1 | F_L;F_G |

a) ***Normalised abundance values are z-scores derived from LFQ intensities following log₂ transformation and missing value imputation in Perseus. Positive values indicate relative enrichment and negative values indicate relative reduction in the indicated condition relative to the global condition mean.***
